# Adolf Nichtenhauser and the history of medical film

**DOI:** 10.1017/mdh.2025.10027

**Published:** 2026-01

**Authors:** David Cantor

**Affiliations:** Instituto de Desarrollo Económico y Social, Buenos Aires, Argentina

**Keywords:** Adolf Nichtenhauser, Medical Film, Health Film, Historiography of Medical Film, Historiography of Health Films, Jewish Immigration from Europe to the United States

## Abstract

The U.S. National Library of Medicine holds two collections by Adolf Nichtenhauser (1903–53) that have become important sources for historians of medical and health films: an unpublished book-manuscript in which he surveys the history of medical and health films to around 1950, primarily in Europe and North America; and the valuable collection of documents he amassed partly during his research for this book-manuscript. Such is the richness of these collections that it is difficult to imagine a history of medical and health film that is not in some way indebted to Nichtenhauser. Indeed, his book-manuscript has become a standard citation in the historiography of medicine, health and film. Yet very little is known about Nichtenhauser himself, other than that he was a European immigrant to the United States who wrote this key history and died before its completion. This article seeks to do three things: to provide the first English-language biography of Nichtenhauser from his early life in Austria to his career in the United States; to use this biography to explain how he came to write this book-manuscript; and to explore the relationship between his historiography and efforts in the 1940s and 1950s to identify and solve problems with application of film to medicine and health.

The U.S. National Library of Medicine (NLM) holds two collections by Adolf Nichtenhauser (1903–53) that have become important sources for historians of medical and health films. One is an unpublished book-manuscript in which he surveys the history of medical and health films to around 1950, primarily in Europe and North America.^1^ The other is the valuable collection of documents he amassed partly during his research for this book-manuscript.^2^ Such is the richness of these collections that a growing body of scholarship on the history of medical and health films is deeply indebted to Nichtenhauser. Both collections have recently been digitized and made available through the NLM’s digital repository.^3^

Nichtenhauser’s unpublished book-manuscript has become an important citation in the historiography of medicine, health, and film since the 1990s.^4^ In it he provides a detailed account of the history of medical and health films, which traces the origins of such films, and their uses as research tools and in medical and public health instruction. It is a broad-ranging narrative that explores the role of wars (especially the two world wars) as turning points in the history of such films; of both national and international perspectives to an understanding of their complex trajectories; of approaching film as a visual language, quite different to that of writing or speech; and of understanding the conditions of production and screening, the histories of film technologies, and the roles of the many groups involved in producing such films: professional and amateur filmmakers, government and private organizations, sponsors, corporations, audiences, cataloguers and archivists.^5^ It is then no small wonder that historians have plundered the book-manuscript’s riches, despite the fact that 177 pages are missing from the NLM’s copy of the manuscript and, at the time of writing, seem to be lost.^6^ Yet, Nichtenhauser’s work does not always get the attention it should. Before the 1990s, very few historians of film used the manuscript because it was relatively unknown and unpublished. But even after his resurrection from the 1990s, some historians omit citations to his work, or (as I shall suggest in the conclusion) use it in ways that do not engage with its major historiographical themes.

Despite such disengagement, recent historiography often echoes Nichtenhauser in highlighting an enthusiasm for film among late nineteenth and early twentieth-century medical and health commentators. In the view of such commentators (and of Nichtenhauser), the motion picture promised to be a transformative technology that would revolutionise professional training, practice, and research and reach mass audiences in ways that other media could not seem to match. Thus, as Nichtenhauser’s book-manuscript documents, from the late nineteenth century, public health officials, medical practitioners, research scientists, and many others turned to film as a new tool of research, for training health workers, and for them to record their routine observations and practices. It also came to be a key part of public health campaigns, incorporated into multi-media efforts against a wide range of conditions including tuberculosis, venereal disease, cancer, and diabetes, to promote the work and ideas of a vast number of health organizations and healers, and to advertise health products such as pharmaceuticals. In short, as Nichtenhauser demonstrates, it shaped the practices and knowledge of scientists, medical practitioners, medical trainees, and students, sometimes in concert with other visual technologies such as the X-ray or the microscope. It also helped to encourage a variety of ideas and behaviours regarding health and illness among the public, and to endorse medical and health institutions and products. The future, it seemed, would be printed on celluloid as much as paper.

But there were also dangers with the new technology. Advocates such as Nichtenhauser argued that the promise of film was often not realised. Too often it seemed that motion pictures discouraged healthy behaviours promoted by doctors and public health advocates, or encouraged more risky ones, and so undermined public health campaigns. Too often they failed to endorse medical institutions and products, or promoted others of which regular medicine did not approve, and so undermined medically sanctioned advertising campaigns. Too often they failed to live up to their potential as educational and training tools and so undermined the training of doctors, public health officials, and other health professionals. And too often they misled even the most experienced doctors and scientists, and so undermined their research and practice.

The reasons for this were much debated. To what extent was it because many films were poorly made or screened, because the technology was not always cheap or widely available, or because of technical limitations inherent in it? Was it a problem with its integration into educational efforts, training programs, and public health campaigns; did the programs and campaigns themselves undermine the value of film, or was it that the films undermined the programs and campaigns? Was it because regular medicine did not have a monopoly on the technology or was divided as to the messages it should promote, so that a variety of films with different, competing messages existed? Or was it because of the audiences it was intended to reach? Frequently, they seemed to respond in quite unexpected and unwanted ways, even to the best made of films. Nichtenhauser’s book was intended to trace the complicated history of film in medicine and health, to highlight its promise for the field, and the roots of then current debates about its benefits and failings.

This paper has three main objectives. First, it seeks to provide the first English-language biography of Nichtenhauser since, despite his importance to the historiography of medical and health films, very little is known about him other than that he was a European immigrant to the United States who wrote an unpublished book-manuscript on the subject. He is sometimes described as a Czech or Austrian (both problematic descriptions given his family roots in Moravia before the creation of Czechoslovakia), and there is occasional recognition in the secondary literature of the problems he encountered as a Jew within the various European territories in which he lived (Moravia, Czechoslovakia, Austria, among others) but there is little on such issues in the United States.^7^ But that is about all there is on Nichtenhauser, apart from a few short pieces.^8^ This article seeks to expand on this literature, situating Nichtenhauser’s life within its sociopolitical context: as a Jew and a left-leaning intellectual in a 1930s Austria increasingly hostile to these groups; and as a left-leaning Jew in a US hostile to communists and ambivalent about Jews. In addition, in the United States his situation was complicated by his position as a migrant unable to secure permanent employment, and as someone who gained a reputation among some of his employers as a difficult figure, unwilling or unable to adapt to their demands. He thus emerges as a paradoxical figure, both central and marginal to the development of medical and health films as a genre and their historiography. In Austria and the United States, he was lauded for his extensive knowledge of medical and health films; his views on how the field should develop were well respected, and in the United States, he gained a reputation as its leading historian. But, in Austria his efforts to establish himself in film and medicine were stymied by the rise of fascism and Nazism, and like many other Jews and left-leaning intellectuals, he was forced eventually into exile. In the United States, his uncertain career path and precarious finances, his difficulties in adapting to the requirements of his employers, and his experience of US antisemitism and anticommunism also mark him out as something of an outsider.

Second, this paper aims to use his biography to explain how Nichtenhauser came to write his history. I suggest it has origins in his long-standing interest in medical, scientific, and public health education films that can be traced back to his early life in Austria, where he developed a career as someone who promoted (and occasionally tried to make) such films, diagnosed problems that hindered their development and use, and proposed solutions; a career trajectory that he continued when he moved to the United States. In particular, his interest in what I shall call film reform – these efforts to identify and solve problems in the production and use of film that impeded their application to medicine and health – would provide a motive for his history which was to help in contextualizing efforts to improve the status and effectiveness of film in the post-war United States. In short, Nichtenhauser’s history of medicine, and the reform efforts in which it was embedded, was shaped by his European origins. This was a book-manuscript that situated US developments within a broader, generally European, history of medical and health film, even as it sought to highlight the unique contributions of the United States to health and medical films.

Finally, this article is about the uses of history in film reform.^9^ Nichtenhauser’s book-manuscript was intended to explore how some of the key problems facing the use of film in medicine and health had been approached in the past and, by extension, how they might be approached in the then present. It thus traces the long history of how hopes invested in film had been undermined: how pioneers of film had seen it as offering valuable means of improving scientific and medical practice and of educating various audiences; and how such expectations had been undercut up to the present day by many issues, including poor production and screening practices and an uneven coverage of significant topics in medicine and health. This story of hope and frustration was, however, little known according to Nichtenhauser. It followed, he suggested, that better knowledge of history could inform current efforts at reform, though (as I shall demonstrate) he was not averse to inserting his own views of the problems of film and their solution into his book-manuscript despite claims that history should not be judged by the present. In short, his book-manuscript can be seen as a form of ‘useful’ history that promoted medical and health films as valuable forms of education and knowledge creation with a unique set of traditions and key figures who established the early use of film in medicine and health, identified problems in their application to medicine and health, and proposed solutions. Film reform, in other words, was nothing new to Nichtenhauser, and there was much to learn from the past.

## Part 1. Twice marginalized

### Europe

The roots of Nichtenhauser’s interest in medical and health films and their history can be traced back to his early life in Central Europe. He came from a middle-class Jewish family from Moravia, a mixed Czech- and German-speaking province of Habsburg Austria and later part of Czechoslovakia, and he sought with some success to establish himself within the worlds of medicine and film. Yet, like too many others, he and his family were to be traumatized by the rise of Nazism. The family would find its property appropriated and careers stymied. Some members would perish in the Holocaust, while others escaped abroad. Nichtenhauser would find that the promise of careers in medicine and film evaporated in Europe. His politics and Jewish identity worked against him, and he was forced to leave the continent.^10^

He was born in 1903 to Hermann Nichtenhauser (1866–1942) and Ida (née Mogyorósy: 1878–1942), the eldest of three brothers, the others being Bedřich (1905–46) and Karel (1908–33).^11^ Originally from Andrychów, Galicia, in 1903, Hermann and Ida moved to Moravská Ostrava/Mährisch Ostrau in Moravia, though Hermann also worked in Vienna, where Adolf (also spelled Adolph) was born.^12^ Moravská Ostrava/Mährisch Ostrau was home to several individuals named Nichtenhauser, some of whom also came from Andrychów.^13^ It is unclear what, if any, relations there were between them.

Hermann was the co-owner of Ecco, an engineering supply company in Moravská Ostrava/Mährisch Ostrau, and two of his sons followed him into the business. Bedřich became a salesman in Ecco after graduating from trade school and owned shares in the Brno branch of the same company.^14^ The youngest son, Karel, worked as a sales assistant for his father also at Ecco.^15^ The family lived a comfortable life, eventually moving to the centre of the town, where many of the affluent middle class lived.^16^ Hermann and Ida visited spas, joined sometimes by their children: Adolf holidayed with Bedřich and their mother at the Hotel Viktoria in Bad Ischl, a small spa town near Salzburg, in August 1932.^17^ A year later, tragedy struck: Karel died in hospital in Moravská Ostrava/Mährisch Ostrau on 14 August 1933. He had committed suicide.^18^

Adolf took a different career path from that of his younger brothers. He obtained his school certificate from the Realschule in Moravská Ostrava/Mährisch Ostrau in 1918.^19^ Then, his CV notes that he studied psychology, art history, and literature at the Universities of Berlin, Bonn, and Heidelberg, and medicine at the universities of Berlin and Vienna: University records broadly support this but give a more complex picture of his education.^20^ He received his *Doctoris Medicinae Universae* (Doctor of General Medicine) on 12 December 1931, from the University of Vienna, after which like other newly minted graduates he practiced medicine in several Viennese hospitals, as internships had to be completed at various clinics and departments.^21^ He also worked as a medical translator.^22^

Nichtenhauser’s interest in film predated his medical career. He himself traced it back to 1923, and a fascination with American silent comedies, ‘which impressed me as examples of true cinematic genius’.^23^ (Nichtenhauser used the term ‘American’ to mean the United States and its peoples, and ‘America’ to refer to the United States, which is the way I shall use the terms.) He began viewing and analysing films, attending trade shows, working as a reviewer for a theatre chain, and claims to have taken courses in motion picture technology in Vienna.^24^ He also reviewed numerous films for various Austrian, German, and Swiss publications, and directed and edited a four-reel documentary on an Austrian youth organization and organized their educational film program, which he claims led him to realize the importance of the 16 mm film technology for the ‘cultural and educational development of motion pictures’.^25^

Austria ‘turning semi-fascist’^26^ changed everything for Nichtenhauser, as the Clerical-Fascist government (1934–38) did for many of his peers, including Social Democrats and Jews, the last of whom had long faced antisemitism deeply ingrained in Austrian and Viennese life. He had begun to establish himself as a writer and critic, notably in the *Wiener Weltbühne*, a weekly magazine for politics, culture, and business. The magazine was an offshoot of the Berlin-based *Weltbühne*, which had been banned by the National Socialists in 1933 and the Vienna publication was barely born when it was itself forced to move its editorial headquarters to Prague, with a new name *Die Neue Weltbühne* for which Nichtenhauser was also an occasional contributor.^27^ ‘Despite the absence of a free press’,^28^ he reflected later, he continued to publish film criticisms and short articles and comments in the daily *Das Kleine Blatt* (Vienna, 1933–34) and the weekly *Allgemeines Illustriertes Wochenendblatt* (Vienna, March–June 1935).^29^ Yet, his writing took him into dangerous political waters. He wrote critically about the impact of Nazism on German film and its implications especially for Austria.^30^ He was dismissed from the General Hospital in Vienna in 1933, according to one commentator, for political reasons (his political writings/activities? Anti-Jewish measures enacted in Austria prior to the *Anschluss* when Germany annexed Austria?) and accused of anti-government propaganda abroad.^31^ It has already been mentioned that he worked in several Vienna hospitals in the 1930s to complete his training in Vienna. But this moving from one position to another was likely compounded by his dismissal, a circumstance not helped by the economic crisis.^32^

Shortly after his dismissal from the general hospital, Nichtenhauser began to publish on a new subject: medical films. Writing in 1934, he lamented the difficult situation of such films in Austria.^33^ In his view, although a significant number were available in Austria, they were rarely screened in part because of a lack of film projectors for the ‘normal’ 35-mm formats. He had become an enthusiast for the 16 mm format as a means of challenging the hegemony of commercial and fascist involvement in theatrical film, and to promote artistic, educational, and documentary film work.^34^ Now, he also saw it as part of a solution to the availability of medical films, and he recommended converting existing medical films to the 16-mm format, procuring other films from abroad, and obtaining more projectors for the 16-mm format. He saw the Österreichischer Lichtbild- und Filmdienst (ÖLFD, 1929/30-8: the Austrian Photographic and Film Service of the Federal Ministry for Education) as a key to reform. It already promoted medical films and Nichtenhauser wanted it to convert some of its medical films to the 16-mm format to demonstrate the advantages of narrow-film technology.

Nichtenhauser was by no means the first to remark on the poor state of medical and health films in Austria, or to promote their reform.^35^ Echoing a common theme among other writers, he argued that Austria had once been a pioneer, but that medical film production had rapidly declined from the early 1930s. There was a need, he claimed, for a more systematic promotion of medical films and the establishment of an institutional framework to deal with visual methods in medicine. He saw film as a way of reducing the need for anatomical dissections and animal experimentation and discussed the value of various formats of film – narrow or normal gauge film (narrow gauge was popular in the United States, but there were problems in its use in Austria), colour or black and white (he preferred colour for some films), and sound or silent (the inclusion of sound in film was not always necessary to him).^36^ He also urged a reform of the economic organization of filmmaking and sales, and the need, as he saw it, for the professional archiving and cataloguing of existing medical films.

Nichtenhauser might have published about medical and health films, but the political situation stymied his efforts to produce such films. Thus, in 1935 he approached the Dean’s Office of the Medical Faculty of the University of Vienna to produce (in collaboration with Selenophon Licht- und Tonbild GmbH) a scientific film called *Oesterreichische medizinische Schule* about the development of the Vienna Medical School. But the proposal was rejected, in part because another production company, Kodak, had already planned or produced a similar film, but also because of Nichtenhauser’s involvement. His earlier dismissal from the General Hospital had come to haunt him. It had been lifted on 1 November 1934, but the School was wary of producing a film – under the aegis of the Federal Ministry for Education and the Medical Faculty – written by someone ‘seemingly incriminated in this respect’.^37^

As the situation deteriorated in Austria, Nichtenhauser began to look for a way out. In 1938, shortly after the *Anschluss*, he left the country, first to Moravská Ostrava/Mährisch Ostrau, Czechoslovakia (where his family still lived), then to Milan, Italy, and then – after some problems in obtaining a visa – to Paris, France, where he repeatedly moved accommodation, often from hotel to hotel.^38^ Now, he sought to exploit his film connections to get to the United States: He had long corresponded with American individuals and organizations about film and had worked as a film critic and published several articles in English-language magazines, including the *Motion Picture Herald*, for which he worked as their Vienna correspondent.^39^ Eventually, he secured a letter from a producer at the Twentieth Century-Fox Film Corporation urging the American consul in Paris to place Nichtenhauser, ‘a man of wide culture and fine background’,^40^ on the quota for entrance into the United States. After receiving a visa for the United States, he travelled from Le Havre to New York on the SS Paris, arriving on 8 February 1939.^41^

### The United States

Like many immigrants to the United States, Nichtenhauser found himself caught between the life he had left behind and the new one he had to build.^42^ He not only had to adapt to his new life in the United States, but carried with him pain of the events that had led to his forced migration, his fears for his family still in Europe, and the financial struggles of an immigrant in an alien world. He started on a series of short-term contracts, but he was never certain whether he would have employment at the end of each. He was constantly changing jobs and short of money: at least one time he had to ask his employers to raise his pay to cover living expenses and to rescue his brother Bedřich from Europe and to care for him when he was sick.^43^ Nichtenhauser also struggled to find acceptance among some of his new colleagues who, as I will elaborate later, found his personality and approach to work difficult from their perspectives, attitudes sometimes coloured by antisemitic suspicion of him as a communist and a Jew. The outsider who had escaped German-Austrian antisemitism had found its American cousin.^44^ As the sociologist, Robert Park, puts it of immigrants earlier in the century, he was ‘not quite accepted’,^45^ marginalized a second time.

Almost immediately after arriving in the United States, Nichtenhauser declared his intention of applying for citizenship. The form describes him as a 35-year-old physician with brown eyes, grey hair, 5′8″, 138 pounds, and with a small scar on the left side of his forehead.^46^ His race is listed as Hebrew and his nationality as German, after the *Anschluss.* (Perhaps because of anti-German sentiments in the United States, he often called himself Nick or Nicky rather than Adolf.) He lived in Manhattan and was unmarried. (He later married Margaret (née Hirsch:1909-2001), another Jewish immigrant from Germany.^47^) He would move accommodation many times, often in the Upper West Side of New York City.^48^ He was joined in 1941 by Bedřich, who would live in New York until his death in 1946.^49^ And he had other family nearby. The manifest of the SS Paris notes he had a cousin, the philologist Dora Nichtenhauser (1890–1968), already living in New York.^50^ And in 1942, Adolf listed as someone who would always know his address, a Lola Nichtenhauser (1892–1982) Dora’s sister, so another cousin, who lived not far away on Broadway at the same address as her sister.^51^ He also petitioned for his parents to come to the United States. His father had been forced to sell his shares in Ecco when the Nazis came to power. He and Ida were transported to Terezin/Theresienstadt in German-occupied Czechoslovakia, and from there to Treblinka where they were murdered on 22 October 1942.^52^ Nichtenhauser’s petition for them to come to the United States was approved two years later.^53^

Nichtenhauser’s work with film continued after his arrival in the United States. He started looking for employment almost immediately, at one point pitching a film idea to Disney (rejected).^54^ As Table 1 shows, he soon found a job with the National Tuberculosis Association as Assistant to the Director of Health Education and then lived on a succession of grants and consultantships. He obtained his citizenship in March 1944, and the following year moved to the Washington, DC, area and began a series of contracts with various Federal agencies which continued until 1950 when he started with the newly formed Medical Film Institute (MFI) of the Association of American Medical Colleges (AAMC).

**Table 1.** Adolf Nichtenhauser’s career in the United States

Dates	Positions	Activities/roles (Adapted from Nichtenhauser’s descriptions)
1939–40	Assistant to Director of Health Education, H. E. Kleinschmidt, National Tuberculosis Association	Advised state and local tuberculosis associations on the selection, use and production of health films; revised film outlines and scripts for them; wrote, directed and edited a film for Bergen County Tuberculosis Association; prepared a text on health film production, and, for the National Health Council, a subject-source list of health films; worked on plan for a central health film agency.
1940	Film Production	Directed the food handler training film, ‘Eating Out’, for the City Health Department, Flint, Michigan (see Figure 1).^1^
1940	Consultant to New York Tuberculosis and Health Association.	Worked on improving health film distribution and programming methods in Manhattan; placed health films in Manhattan movie houses; conducted audience tests with health films.
1941–45	Section on Health and Medical Films, American Film Center (funded by a grant from the Division of Medical Sciences, Rockefeller Foundation).	Prepared film catalogues and critical film lists; analysed about four hundred films^2^; developed health film evaluation methods, carrying out reviewing projects with or for agencies including the Office of War Information, New York City Nutrition Program; conducted a national and international information and consulting service on all aspects of health and medical films; prepared film analyses for producers and advised government and private agencies on planning of production programs; prepared and organized conferences, to advance plans for a medical film institute, in conjunction with the annual meetings of the American Medical Association (1942) and American Public Health Association (1943)
1943–44	Grant from the National Science Fund, National Academy of Sciences.	Revised and reedited the twenty-reel film series, ‘Neuropsychiatric Disorders’ by S. Philip Goodhart and Benjamin Harris Balzer
1945	Office of War Information, Bureau of Motion Pictures.	Prepared recommendations for the coordination and improvement of the health and medical film activities of seventeen Federal agencies.
1945	The Bureau of the Budget, Work Simplification Section.	Outlined measures to establish optimum standards of procedure in government film production, distribution, and utilization.
1945	Department of State, International Motion Picture Division.	Prepared a study of the objectives of an international cultural film program and its implementation by an International Film Institute.
1945–46	Visual Information Specialist, U. S. Public Health Service, Malaria Control in War Areas, Training and Education Division, Atlanta, Georgia; and Office of Health Information, Audio-Visual Section, Washington.	**Atlanta:** Prepared recommendations for reorganizing production and distribution activities; prepared catalogues of films distributed by office; reviewed films for use in training courses; produced, researched, and scripted an entomological training film; participated in planning for other productions. **Washington:** Prepared recommendations on general film activities of the Public Health Service, especially on distribution policies and methods; production research and outlines for two films on rabies control. As Public Health Service representative on the Interdepartmental Special Committee on UNESCO Problems, participated in reviewing 150 American educational films; selected health and medical films for UNESCO; and prepared film notes and program suggestions.
1945–46	Consultant to Federal agencies (on loan from U.S. Public Health Service).	**Army, Lawson General Hospital, Orthopedic Division**. Planned production procedure for a series on the Second World War amputation techniques and prosthetics. **U.S. Department of Agriculture, Farm Security Agency, Health and Medical Services.** Planned and outlined a film on rural health. **Veterans Administration, Department of Medicine and Surgery, Medical Illustration Service.** Recommendations on scope and organization of medical film production program.
1946–47	Contract work for the Department of State, International Motion Picture Division, Washington.	Prepared evaluations and recommendations of about 200 films for worldwide medical film distribution program.
1947–50	Consultant to the Navy Department, Bureau of Medicine and Surgery, Audio-Visual Training Section, Washington.	Worked on the post-war reactivation of Navy medical film activities; analysed production outlines; wrote, directed, and edited a short film on the acrylic eye; prepared ‘A History of Motion Pictures in Medicine’.
1950–52	Staff Member, Medical Film Institute (later Medical Audio-Visual Institute) of the Association of American Medical Colleges, New York.	Developed principles and practice of medical film reviewing, working with panels of subject-matter specialists and with test audiences; trained physicians and scientists in film analysis and evaluation techniques; supervised preparation of nearly 200 film reviews (on grants from Rockefeller Foundation, National Cancer Institute and National Heart Institute); in charge of Department of State contracts to select medical films for use abroad and prepare information on their content and utilization.

1 ‘Austrian Doctor Here Directing Film Tells of Foreign Movies’ (8 February 1940), *Flint Journal*, Cutting in Sloan Museum of Discovery, 221 E Kearsley St, Flint, MI 48503, United States.

2 Some of these analyses are in File, ‘Rockefeller Foundation’, Nichtenhauser Collection, NLM. **Source**: Adolf Nichtenhauser, ‘Curriculum Vitae’, April 1952, Nichtenhauser Collection, Folder, ‘Nichtenhauser, Adolf – biographical’.

**Figure 1.**
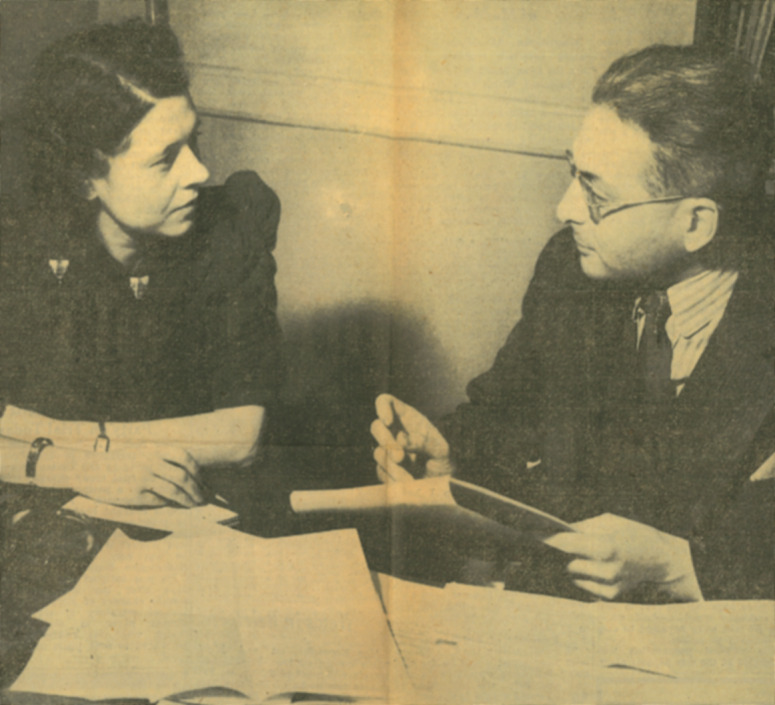
Adolf Nichtenhauser made several educational films after moving to the United States (Table 1). Here, Nichtenhauser and Evelyn Clemens (Secretary to Dr. George Hays, City Health Director, Flint, Michigan) are photographed with the script of the city health department’s educational motion picture *Backstage with the Food Handlers*, released as *Eating Out* in 1940. *Eating Out* was a 16-mm two-reel silent movie intended to educate food handlers about disinfection techniques and hygienic methods of handling food. It was produced by Nichtenhauser under the supervision of H. S. Adams, Director, Division of Food and Sanitation, Flint. **Source:** ‘Austrian Doctor Here Directing Film Tells of Foreign Movies’, *Flint Journal* (8 February 1940). Cutting from the Sloan Museum of Discovery, 221 E Kearsley St, Flint, MI 48503, United States. © 1940 MLive Media Group/The Flint Journal. All rights reserved. Used with permission.

The last appointment took him to the heart of post-war efforts to improve the quality of medical and health educational films. But it was a long time coming, and in part because he had gained a mixed reputation among colleagues and employers. Many valued his knowledge of medical and health films, but not all, and opinions on him could be harsh. In 1945, John Grierson (Commissioner, National Film Board of Canada [NFB]) turned down a request to hire Nichtenhauser for 3 months because he found him ‘an intolerable bore’^55^ and felt that the NFB ‘would lose much kudos’ if it employed him. The same year, Alan Gregg (Director, Division of Medical Sciences, Rockefeller Foundation) informed Nichtenhauser that he was ‘not prepared’ to provide a recommendation for a grant ‘of your all around capacities since it seems to me that you still have a good deal to learn in point of dealing with people’.^56^

Grierson’s and Gregg’s comments likely had roots in Nichtenhauser’s problems with his 1941 appointment as head of a special section of the American Film Center (AFC), funded with a three-year grant from the Rockefeller Foundation.^57^ The AFC had been established in 1938 by the Rockefeller Foundation to promote the production and use of documentary and educational films, and the special centre was to focus on the use of motion pictures in health education and in medicine and to act as an information and advisory centre in these fields.^58^ Yet, his employers were wary of Nichtenhauser. He had tremendous knowledge of medical film, but they found him to be awkward at meetings, often alienating those whom he should be cultivating. Donald Slesinger (1897–1977), Nichtenhauser’s boss at the AFC, felt that his personality ‘stood in way of satisfactory dealing with various medical and public health groups’^59^ and that there were some problems with the accounts: ‘N. didn’t get on well with people and insisted on maintaining tight central organization instead of developing resources in the field as S.[Slesinger] would have preferred’.^60^ For his part, Nichtenhauser complained that Slesinger was not ‘a competent man in this field’,^61^ and eventually things came to a head. At the beginning of 1945, he was suddenly fired, the announcement made to an unsuspecting Committee on Films of the Conference of Teachers of Preventive Medicine: ‘Consternation in meeting when N’s dismissal announced’.^62^

Things were also difficult in another job when this refugee from Europe, whose parents had been murdered by the Nazis, became the subject of antisemitic and anti-communist attacks in his new country. In one 1948 encounter, Nichtenhauser claims a Captain Robert V. Schultz, angrily informed him that ‘All Jews are Communists. For 2000 years the Jews have caused trouble. Always working against Government. They always want something new, don’t let things as they are’.^63^ Schultz was the head of the U.S. Navy’s Audio-Visual Training Section, Bureau of Medicine and Surgery, and had hired Nichtenhauser in 1947. Schultz also suggested that Nichtenhauser stole others’ work, parading it as his own. He also violently objected to the inclusion of a citation to Nichtenhauser’s history into a document presented under Schultz’s name, so violent that, according to Nichtenhauser, ‘Dr. Schultz became incensed and said that he would strike me’.^64^ Nichtenhauser reports that he said nothing in reply, ‘at which he [Schultz] leaned over, took off my glasses and threw them over on his desk’.^65^

Such events likely had uneasy echoes for Nichtenhauser of the problems that had blighted him in Austria, and given the uncertainties of his career in the United States – jumping from one short-term position to another, not unlike his medical career in Vienna – this twice-marginalized man would have wanted to avoid a repeat of the difficulties he had earlier experienced. So, in public he said nothing about his troubles with Schultz. On the contrary, he tended to heap praise on his boss, crediting him with the beginnings of his book-manuscript. Schultz had originally contracted Nichtenhauser to produce a monograph on the general status of medical films.^66^ According to Nichtenhauser, however, after Schultz read a draft of the manuscript, he became convinced that much of what happened in the past was so significant to then-contemporary endeavours that a historical account would help to understand them better.^67^ Nichtenhauser might have come into conflict with Schultz in the office, but he avoided such a conflict in his history.

## Part 2: Nichtenhauser’s history

### The prisoner

As Nichtenhauser’s comments above suggest, his book-manuscript was intended to contribute to efforts to reform medical and health film production, distribution, and evaluation after the war. There were thousands of such films, but Nichtenhauser – and other contemporary film reformers – saw them as varied in quality and effectiveness. Many were poorly produced, but even the best could have quite the opposite effect on audiences to what was desired. In addition, coverage was uneven: some fields of medicine and health were overrepresented in film, while others were neglected. And to complicate the issue further, there were structural issues of poor distribution, funding, and screening practices. The Navy, which employed Nichtenhauser, had invested heavily in film as a tool of education during and after the war and wanted to ensure its effective use.

The reformist impetus behind the book-manuscript did not mean, however, that he saw his history as being tightly guided by current concerns. It was essential, according to Nichtenhauser, to undertake research with an open mind if the historian was to fully understand the roots of current issues and approaches. Historians might have to wander far from current concerns to fully understand the past and how it shaped the present. Nichtenhauser, the historian, had to follow his instincts and sources (wherever they led), and immerse himself in his subject, so that he could return to the present with new insights. Film’s past could not be judged according to contemporary values or ideas. Rather, it had to be understood in its own terms. His history was to distance itself from judging the past. Facts had to be presented objectively and dispassionately, a quasi-Rankean view of history that also allowed him to avoid any commentary likely to arouse unwanted attention from his government employers.

In Nichtenhauser’s view, historical research was a constant source of amazement and unexpected findings, which opened new understandings of present-day practice. It revealed some practices to be older, or more (or less) widespread, than hitherto realized. It led to the discovery of previously unknown developments, or paths taken and subsequently abandoned. It might in the long-term provide results of practical value to contemporary filmmakers and sponsors, but there was no guarantee that it would. Without plunging into the messy unpredictable past, without abandoning preconceptions of how things were, and without taking the risk of following historical paths that might go nowhere – without all of this, one would never fully know how the past shaped the present, though it remained a possibility that the absence of political commentary would undermine this goal.

So, Nichtenhauser found himself grubbing around for more information to fill in his story and came to love the past for its own sake as much as for its reformist purpose. He writes of being a ‘prisoner of his subject’,^68^ and of the pleasures of rooting out unknown stories buried within the professional published literature and in archives and interviews. He tells of the surprise of uncovering ‘long-forgotten facts’^69^ and of how this leads to more research. And he mentions the difficulties of obtaining copies of films, the problems of illustrating these films in his book-manuscript, and of making copies available for the reader to view. One suspects that Nichtenhauser not only found himself imprisoned by his subject but also liberated by it, free to follow whichever byway his research took him, and often to places that had little bearing on then current concerns. It was likely that in part for this reason his book-manuscript was slow in coming, and perhaps also part of the reason why Schultz became frustrated with him. Schultz wanted the book-manuscript published, but Nichtenhauser stalled, resisting efforts to cut the length, and continuing research and writing almost up to his death.^70^

### Themes^71^

Nichtenhauser’s scouring of the many byways of medical film had a broader historiographic purpose. He wanted to tell stories that were complex, multifaceted, full of different ideas and opinions and different routes and outcomes, and his search for obscure facts was part of this effort to do this. He constantly added more details to complicate his story, and if this complexity took him away from the immediate concerns of medical film in the 1940s and 1950s, it also allowed him to capture the contingencies of past developments that few histories had explored before. In Nichtenhauser’s telling, the history of medical and health films was multilayered, went in numerous directions, and often ended in different places.

None of this complexity, however, was to be at the expense of broader themes that helped explain the development of medical and health film. One of these themes concerned the role of war – especially the two World Wars – in shaping the development of medical and especially health films.^72^ In Nichtenhauser’s account, it was during these wars that the health film gained a mass audience, increased funding, and important roles in a variety of public health campaigns, even if such funding was sometimes short-lived as he suggests was the case in the United States, France, and Germany after the First World War – a forewarning perhaps of what he feared might happen after the Second World War.

The last point highlights a second theme for Nichtenhauser: the importance of national contexts.^73^ While much of the then existing historiography of medical and health films focused on developments in one country or another, it generally did not systematically trace their different trajectories in different countries: the country was just the location where things happened. Nichtenhauser’s book-manuscript challenged such stories by showing how national contexts mattered. The development of medical and health film was very different in Germany, France, Great Britain, and the United States, the main foci of his study, though he also highlights the importance of international organizations (especially after the Second World War) in attempting to bring order to this hotchpotch of national developments.^74^

A third related theme is the interconnected roles of government and commerce. In the case of the United States, for example, Nichtenhauser argues that commercial organizations took little interest in medical and health films before the First World War and that state and community organizations’ involvement preceded that of the Federal government.^75^ All this changed with the war. The Federal government became enthused by the idea of film as a tool of research and education, and helped to turn around commercial reluctance to support non-theatrical films.^76^ But the enthusiasm of both commerce and government diminished when hostilities ended.^77^ Commercial organizations filled some of the gaps.^78^ However, they were selective in what they would support,^79^ and their interest fluctuated with economic conditions: even when health film production recovered after the depression of the 1930s, US commercial filmmakers remained hesitant about the market, a key exception, Nichtenhauser suggests, being the *March of Time*, a newsreel series sponsored by Time Inc., screened in movie theatres between 1935 and 1951.^80^

Nevertheless, commercial interest in film was not without its problems. He documents several issues: controversies around the transformation of VD educational films into risqué commercial entertainment products;^81^ distrust of some commercial film producers and their involvement in medical cinematography;^82^ and the questions about whether commercial organizations always made more effective films.^83^ Government involvement was also complicated. It became involved in visual education after the First World War,^84^ cooperated with or competed against commerce,^85^ hired commercial companies (such as in the United States) to make educational and training films, and facilitated the introduction of Hollywood techniques into medical film.^86^ However, while in Great Britain and Canada governments produced public education films on wartime problems, in the United States, production was more complicated with some agencies getting government funding and others not.^87^ In short for Nichtenhauser, there was a complicated relationship between commerce and government that varied over time and from country to country.

Several other themes were also important to Nichtenhauser. First, he highlights the various categories of films that make up the subject of his book-manuscript: ‘medical cinematography’ as he calls it.^88^ For example, the broad category of medical cinematography included the subtype of the public health education film, or ‘health film’ as Nichtenhauser calls it, which itself included a variety of sub-subtypes: the ‘dramatized health film’,^89^ which emerged in the 1910s; or the pre-First World War films of record of the US Public Health Service (‘not organized teaching films but merely records of the many-sided activities of the Public Health Service’^90^) or the hybrid cross between the popular science film and the health film (an example here being a film about the life cycle of the fly, the first health film Nichtenhauser claims).^91^ In Nichtenhauser’s telling, these different types and subtypes of film allowed him to explore medical cinematography as comprising various forms of film, each with its own history, in turn shaped by the national contexts in which they developed, though sometimes the trajectories of each history are difficult to follow, buried under the details of his story.

Second, he is concerned with the relationship between the development of film and the histories of science and technology. His story of the origins of film, for example, is in part a story of the relationship between the history of film technologies and the history of ideas of and research into visual perception.^92^ And later, there are stories about the relationship between film and the biological and clinical sciences – he explores, for example, the role of film in extending the ability of observers to observe, by slowing down the action, speeding it up, magnifying it, capturing the uncommon or passing; allowing people to go places hitherto difficult or impossible.^93^ This is a story of the technologies of film production and screening, often in combination with other technologies or practices such as X-rays, tissue culture, or surgery.

Finally, he is also concerned with much more than film production, the focus of much historiographical attention before this book-manuscript.^94^ To Nichtenhauser, sponsors and funders were also important to the history of medical cinematography, as were those who distributed films, and screened, collected, catalogued, archived, evaluated, and viewed them: the audiences. His book-manuscript is, in part, an effort to understand these different perspectives and the complicated interactions between them, how each perspective developed differently in different national contexts, how they were interrupted and shaped by war (and peace), and how they shaped different types of film – medical, health, scientific, and so on. He is not always consistent in his approach to telling such a complex story, and the stories can get lost in the mass of information he provides, but his commitment to telling it helps explain why his history took so long to write.

### Reform

Yet, for all his efforts to focus on historical themes, he never escaped the reformist impulse. His book-manuscript is in part an account of the longer history of concerns about the quality and effectiveness of films and of earlier reform efforts, including issues with which he himself had been involved.^95^ One example of this is the book-manuscript’s discussion of his long-standing interest in 16-mm film as an alternative to the standard 35 mm. It traces the development of 16 mm medical and health films in the United States and how they changed the conditions of filmmaking. And it explores its later development in Germany (though not Austria, where he had first raised the issue).^96^ Before the war, he had hoped that 16-mm film would help amateurs enter the field and the book-manuscript documents this for the medical and health fields.^97^ But whereas in the 1930s he had tended to see amateurs as a chance to break commercial and political strangleholds on cinema, in the 1940s and 1950s he came to see limitations to their involvement in film. Alongside discussions of the rise of amateur filmmaking, the book-manuscript is full of accounts of ‘the cinematic horrors’^98^ produced by such filmmakers.

Such comments about horrors not only point to how Nichtenhauser sought to trace the longer history of reform efforts, but they also highlight how he also used it as an opportunity to smuggle in his own critique of medical and health films under the guise of history; this despite his view that the past could not be judged by the present. In the horrors quote above, he simply inserts his own assessments into the narrative. At other times, he seems to promote his own assessments by using the voice of his actors to promote complaints close to his own views of the problems of film. Actors’ concerns about ‘amateurish’^99^ filmmaking reflect his own ideas concerning the impact on film of bad camera work, lighting, and acting, inaccurate and out-of-date content, compounded by issues he had long identified as problematic: the poor conditions under which films were often screened, for example, or the malign interests of some commercial filmmakers or advertisers, or the difficulties in distribution and supply that forced health workers to take any film regardless of quality or physical condition.

This blurring of the voice of the historian and historical actor is there in many of Nichtenhauser’s discussions of technical innovations in filmmaking. Take, for example, his account of the introduction of sound into filmmaking after 1927 and its relationship to the use of film in teaching.^100^ Nichtenhauser uses this to highlight the concerns of his historical actors about the introduction of sound: how it proved a challenge to film as a visual story-telling medium (a reflection of his own views, as I shall document later), the technical difficulties of screening sound films in medical institutions,^101^ or whether sound should replace the teacher.^102^ At times, it is difficult to know whether the views expressed are Nichtenhauser’s or those of his actors.^103^ At other times, he does not hesitate to insert his own evaluations of the impact of sound;^104^ ‘the great curse of the American non-theatrical sound film: “the illustrated talk”’;^105^ the addition of ‘thoughtless verbosity to thoughtless filming’;^106^ his assessments that verbalization weakened film’s visuality, ‘the very essence of the motion-picture medium’.^107^

All this is brought together in the final chapter of the book-manuscript – ‘The Lesson of the Past and the Task of Present’ (available in outline only) – which seeks in part to explain why film had not, by the 1950s, been fully taken up in medicine and health education. It is here that Nichtenhauser’s voice finally emerges from the shadows of those of his historical actors. He argues that his book-manuscript has shown that film is neglected in part because of bad amateur filmmaking, ‘film-illiteracy’,^108^ including failures to understand film as a visual medium among other factors such as the lack of projectors, an old concern of his, as I’ve noted above, dating back at least to his concerns about the status of medical film in Austria. Crucially, he argues that neglect is a result of conservatism on the part of ‘the collective human mind’,^109^ by which he means an unwillingness to abandon ‘verbal thinking’.^110^ Film required a new way of thinking, teaching, and communicating: a new form of language.

### Visual language

The last point highlights a further issue important to Nichtenhauser’s view of the quality and effectiveness of films: the visual language of film. As he put it, the problem of filmmaking was not primarily ‘one of equipment, photographic technique and finances but one of thinking in a visual language which has its own particular grammar, syntax and logic’.^111^ Nichtenhauser had been interested in this subject since the 1920s, when the ideas of theorists such as Vachel Lindsay, Ricciotto Canudo, Louis Delluc, and Béla Balázs had circulated in the Vienna film circles that he frequented. (Indeed, Balázs had written his pioneering work on the subject, *Der sichtbare Mensch* [1924; *The Visible Man*] while living in exile in Vienna [1919–26] about the time that Nichtenhauser claims he first became interested in the subject.^112^) Nichtenhauser makes no mention of these theorists, though some of their ideas on the differences between thinking and learning with words and with images may be there.^113^ Nor does he comment on the related history of the ideas or practices of visual education, despite the fact that Otto Neurath, one of the pioneers of the subject, had also overlapped with Nichtenhauser in Vienna.

Instead, Nichtenhauser tells a more personal story, tracing his interest back to his discovery of American silent comedy in the 1920s. In his account, these movies had established a visual repertoire that distinguished them from earlier forms of storytelling, allowing filmmakers to create narratives without the use of words, except for the inter-titles. Yet, he soon came to worry about the future of this language, fearful that the introduction of sound and the commercialization of cinema could undermine the primacy of the visual.^114^ On the negative side were the sound films of Buster Keaton. Nichtenhauser notes that Keaton’s silent films had been major works of visual art, rich with cinematic and theatrical design, visual parodies of modern American civilization, but his artistry had largely disappeared by the 1930s as the industry forced him to abandon silent film and the visual creativity it allowed.^115^ On the positive side were the sound films of Harold Lloyd. Nichtenhauser loved Lloyd’s *Movie Crazy* (1932 – German-language version *Der Kinonarr* [1932]), a talkie-comedy that Nichtenhauser enjoyed for its continued use of visual techniques typical of silent films to tell a story and make an audience laugh.^116^ In short, the introduction of sound into entertainment films was only a problem where it – or the commercial interests of filmmakers and theatre owners – undermined visual creativeness.^117^

In the case of medical cinematography, especially educational films, Nichtenhauser came to see related problems with sound. He noted that the internal coherence and logical development of a theme within a film should not rely on the narration often used in educational and documentary films. Nor should it rely on that other common feature of medical and health education films, a lecturer or speaker who explained to an audience what was going on screen and answered questions and concerns.^118^ Too many health education films were little more than illustrated talks, he claimed. Too often, audiences heard people talking on the screen instead of seeing what they were talking about. ‘The greatest asset of the motion picture medium is its power of visualization, that is, its power to show things, people and actions in a specific and intense way’,^119^ he explained in one 1943 presentation. Physicians, public health officials, and others interested in medical and health films, he argued, had to learn a new visual grammar and syntax.

Yet, many medical filmmakers failed to understand this. If a problem in entertainment films was the commercialization of the industry, in medical films it was the dominance of amateurs. As Nichtenhauser later put it in his book-manuscript, medical filmmakers were often ‘unaware of the fact that what they were doing was speaking in a new and specialized visual language without command of its orthography and grammar’.^120^ Like amateurs more generally they often did not know basic things about filmmaking. They over- or under-exposed the film, failed to adequately illuminate what they shot, and often handled the physical film badly, further undermining the quality of the visuals. Then there was the question of how to create visual flow. Too often, he argued, amateurs did not understand how to connect separate shots in a sequence or to transition between them. For example, they did not alter the camera position between shots and so failed to create visual interest or to allow the viewer to see different aspects of an object or action. In scientific films, they often failed to insert a title or a dissolve to indicate a lapse of time. Such issues made a case for increased commercial involvement in filmmaking, since commercial filmmakers tended to employ professional filmmakers less likely to make such basic errors.

The commercialization of the film industry was thus a mixed blessing to Nichtenhauser. On the one hand, in entertainment films it posed a threat to visual creativity (a common theme among early twentieth-century German-language commentators^121^), though it was not an inevitability as his comments on *Movie Crazy* suggest. On the other hand, in medical cinematography commercialization could rescue visual creativity from the dead hands of the amateur, though again this was not an inevitability – some amateurs made visually interesting films, while commercial filmmakers could undermine such efforts. But overall, commercialization pulled in two different directions: a threat to visuality in entertainment films and a saviour in medical cinematography. His views on the amateur and commerce had changed since the 1930s, when the former was to save films from the corrupting influence of commerce.

### Audiences

A final issue important to Nichtenhauser’s views of the quality and effectiveness of films concerned audiences. The book-manuscript highlights long-standing concerns that films were ineffective because filmmakers either paid too little attention to audiences (despite a growing body of audience research from the 1920s^122^) or because their films were directed at the wrong sorts of audiences. A film that was targeted at the mass audience did not necessarily work for a more specialized audience;^123^ one targeted at adults might not work for children.^124^ In addition, there was also worry that filmmakers paid too much attention to scientists in public health films; as he quoted one commentator, health agencies should ‘view a picture not from the standpoint of the scientific man but from that of the public which is to see it’.^125^ In short, there were multiple audiences for medical cinematography.

Nichtenhauser’s argument about multiple audiences had roots in his pre-war concerns that commercial filmmakers were primarily interested in targeting mass audiences.^126^ In his view, there was nothing intrinsic to film that meant it could only be shown to such audiences. It was possible to imagine a world in which films were targeted at smaller, niche audiences, much like books or audio records.^127^ It was here that medical cinematography was important, for it seemed to demonstrate to the 1930s Nichtenhauser the many different audiences for film that commercial filmmakers ignored at their peril, and his book-manuscript later picked up on the theme. The 1950s manuscript documents the varieties of audiences that medical cinematography appealed to, how they changed over time, and were dependent on national context and the types of film concerned – medical, health, and scientific movies and their various subtypes.

The book-manuscript’s argument about the multiple audiences for film might have had roots in the 1930s, but some of the concerns that had motivated him then seemed to have changed. His pre-war criticism of commercial cinema’s focus on mass audiences was more muted by the time he wrote the book-manuscript, and his anxieties about mass culture had also changed. Like many pre-war commentators, the 1930s Nichtenhauser had worried that mass audiences were vulnerable, easily manipulated entities, lacking the will to resist; characteristics cultivated by commercial film producers and, more worryingly, by the Nazis. Austria was dominated by German film production, he argued in the 1930s, enduring an ‘entire flood of mud from German film production’, a ‘soul poison [*Seelengift*]’, which was ‘not least to blame for the torpor and irresolution of large masses of people’.^128^

The postwar Nichtenhauser, however, seems less concerned about such vulnerabilities, a strange shift given growing concerns in the 1950s about what came to be called brainwashing.^129^ On the contrary, in his book-manuscript, he seems at times to come close to blessing the idea of manipulating audiences. Thus, he approvingly quotes one commentator about a 1927 film as ‘so full of sensations and exciting catastrophes that the public hardly realizes that it is being informed and instructed. For this very reason the effect, because unconsciously absorbed, is all the more lasting’.^130^ He also approves of the words of another commentator that ‘It is infinitely better, nine times out of ten, to instruct the general public without giving it the impression that it is receiving a lesson’.^131^

## Part 3. A European Jew in America

It should be clear from the foregoing that Nichtenhauser’s historiography was shaped in part by his European background. He had a deep knowledge of German-language film, had developed extensive contacts elsewhere in Europe, and his efforts to escape the continent allowed him to expand his knowledge of European developments further. He explained, for example, that when he arrived in Paris awaiting his visa for the United States, he took the time to learn more about French film, and this later found a place in his book-manuscript.^132^ Equally, his long-standing correspondence with individuals in Great Britain, the United States, and other countries gave him opportunities to write a comparative history of medical cinematography, and his papers at the NLM show that he continued this correspondence as he researched his history.

In Nichtenhauser’s view, few American historians had such a broad knowledge of medical cinematography. Things were changing with the recent influx of European scholars including Nichtenhauser, but an older American historiography was still influential, quite insular in outlook, rarely stretching beyond the Anglo-Saxon world, except to discuss technical developments devoid of local context. Few of those writing the older American historiography had knowledge of languages other than English, which in Nichtenhauser’s view was part of a broader post-war suspicion or hostility to the foreign or unfamiliar. His history can be seen as a counter to this. It sought to foster international exchange and conversation, to open minds to the foreign and unfamiliar. It presented developments abroad to a US audience and situated US developments in international contexts. Perhaps too it had a more precise audience: Robert V. Schultz, the man who had commissioned the history, but who also embodied the prejudice he encountered in his new country.

Yet, there was a risk in pushing the foreign and unfamiliar. Nichtenhauser had learned from his experience in Austria that unwelcome views could have dangerous political consequences, and the uncertainties of his career in the United States likely made him cautious about pushing too far. Government practice was to conduct internal reviews of publications as checks on their quality and emphasis, and anything deemed politically or otherwise problematic could be tricky. He felt he was already regarded as suspicious by Schultz, whose anticommunism and antisemitism were uncomfortable reminders of his time in Nazi Europe; reminders given weight by Truman’s 1947 executive order to screen federal employees for possible association with totalitarian, fascist, communist, or subversive organizations. While Nichtenhauser was not an employee, the surveillance extended to contractors such as himself. So, he retreated into a quasi-Rankean collection of neutral historical facts, and lots of them.

Such caution in his writing contrasts with the conflict he sometimes came into with his employers. He regarded Donald Slesinger as unsuited to promoting the reform of educational film and disagreed with him about the direction of his unit. He also came into conflict with Schultz over the latter’s unwillingness to cite him, his pressure to cut the length of the book-manuscript and to get it published; disagreements heightened by what Nichtenhauser saw as the provocations of Schultz’s antisemitism and anticommunism. He gained a reputation as unnecessarily blunt in his criticism of his employers and socially awkward. As Alan Gregg put it, Nichtenhauser had yet to learn how to deal with people. He did not bear fools lightly, and as such, could be his own worst enemy in institutional hierarchies including the military where he did not hide his disdain of those above him.

There are thus three related paradoxes to consider regarding Nichtenhauser. One is that he was both cautious and confrontational with those around him; politic in his writing but indiscrete or awkward in some of his personal interactions. He seems to have been most comfortable with those he regarded as his intellectual equals or betters. His correspondence in the NLM collection is testimony to the vast communication network he developed, and with which he often engaged in intellectual debate. For example, in 1946, David Ruhe (his future boss at the MFI) highlighted a ‘considerable disagreement’^133^ with Nichtenhauser’s ‘emotional fixation’^134^ on film criticism and his failure to give sufficient attention to whether films worked as teaching devices: ‘I am not at all certain in my own mind that an excellent film from a film critic’s standpoint is an excellent teaching film at all. I am not sure that many films which lack polish, and were very inexpensive, and were even very amateurish, have not been in the long run effective teaching media’.^135^ In general, Ruhe found Nichtenhauser too focused on film production, when the focus should be on education. Nichtenhauser disagreed with Ruhe, but not in the way he disagreed with Slesinger or Schultz. In his mind, it was an exchange of views of two equals.

A second related paradox is that while his correspondence network is revealing of his intellectual debates with and debts to colleagues, he tended to hide such influences on him in his unpublished manuscript. This silence may in part have been related to his efforts to avoid difficult (political) subjects, but often the reasoning is less clear.^136^ The result is that he can emerge as a *sui generis* figure in his book-manuscript, unique in his own ideas and approaches, even as he also sometimes uses the ideas of his actors to promote his own. This is not the place to undertake the herculean task of unpacking the myriad influences on his writing, but as I have indicated throughout this paper, his comments in the book-manuscript often engaged with broader social and political debates. He was, for example, far from alone in his view that film could influence mass publics vulnerable to the siren calls of fascism, Nazis, and commercial organizations. Nor was he alone among immigrant scholars in his anxieties about the narrowing of the American mind in post-war United States.^137^ He is anxious in his history to trace the roots of ideas and practices in the history of medical film, but not so anxious to trace the roots of his own ideas and practices, beyond the citation of primary sources.

The third paradox of Nichtenhauser is that he was also both central and marginal to film reform and its history. He was central in that his wide knowledge of film in many different countries was valued by colleagues: He helped establish some of the major initiatives to reform film in the country, evaluated many hundreds of films, came to be a key part of the MFI, one of the major initiatives in film reform, and his history was regarded by reformers as perhaps the most comprehensive account of the development of medical and health film available. But Nichtenhauser was also marginal given his uncertain career path, short contracts, problematic finances, the incomprehension and hostility he sometimes encountered, and likely his difficult personality. He might have escaped Nazi Europe, but he was unable to fully escape its consequences. He lost his parents and helped his brother out of Europe and cared for him in the United States. He remained in part a European Jew even as he sought to build a new life in the United States, never quite willing or able to break with his past world, and not quite accepted in his new one.

Nichtenhauser’s history also suffered a mixed fate during his lifetime. To motion picture enthusiasts, it offered a unique perspective on key issues in the field: the revolutionary promise of film for medicine and health, and why it had sometimes failed to live up to this promise. No other account of the subject came close to providing such a wide-ranging interpretation of the complex development of the field, the hopes invested in it, and past efforts to explain and solve its failings. As such, the book-manuscript could have become an important contribution to contemporary efforts to promote and reform medical cinematography and so ultimately also help to guide the transformation of medicine and health through this medium. Yet, this did not come about. The history languished in typescript, incomplete, without a publisher, and rarely read except for a few cognoscenti. Likely, some readers used ideas and themes in history to push for reform or promote the potential of film, but the book-manuscript did not gain a wide audience in the 1940s and 1950s, even if small parts were published as stand-alone articles. Its promise was thus not realized while Nichtenhauser was alive, a parallel to the unrealized promise of its subject (medical cinematography) and perhaps of its author.

Nichtenhauser’s own voice went silent too early. He died in November 1953, aged 50, and was buried in Queens, New York, leaving the manuscript unfinished. The Office of Naval Research and MOMA consulted with Margaret, his wife, and appointed an unnamed editor, a self-described ‘close associate and student’^138^ of Nichtenhauser, who worked on the typescript as his main job allowed. But his time was limited, and while the manuscript was revised and edited, it still did not find a press willing to publish it. It seems to have circulated among a few government and MOMA administrators and remained alive in the memory of those who had worked with him and to the few scholars who knew of his work, its audience shrinking as the initiates who knew of the book-manuscript dwindled. So, it came to be read less and less in the years after Nichtenhauser’s death, buried at MOMA and in various government agencies until its arrival at the NLM in 1981, where it joined an earlier donation of his research papers, files, and correspondence.^139^

It was only after the arrival of the manuscript at the NLM that its resurrection began. Suddenly, it was more easily available and, together with the associated archival papers, it has helped to foment, over the last 35 years or so, a growing scholarly interest in the use of film in medicine and health. Yet, in some ways, Nichtenhauser’s historiography remained sidelined. Until their recent digitalization, the use of the manuscript and papers involved a trip to Bethesda, Maryland, where the papers and manuscript were (and are) located, and not every scholar was able to make the trip or was aware of the importance of this collection.^140^ In addition, even those who did use Nichtenhauser’s manuscript tended to make only limited use of it. Instead of engaging with his broader historiographical arguments, the tendency was and is to use the manuscript as little more than a source of information or context on certain medical and health films or types of films, or on individuals, events, and technologies.^141^

It is a puzzling neglect, since Nichtenhauser’s desire to develop a complex, multifaceted history, full of different ideas and opinions and different routes and outcomes, anticipates approaches of many contemporary historians. Equally, his suggestions about how such a history might be achieved find echoes in contemporary historiography: his beliefs that the history of medical and public film must take account of national, international and transnational contexts; that it must go beyond conditions of production to explore of the sponsoring, screening, archiving, and collecting of films; that the history of film is about much more than the history of its technologies and finances, but is a story of the emergence of a new form of visual language; and that the historian must highlight the multiplicity of groups involved in such films: professional and amateur filmmakers, government and private organizations, sponsors, corporations, audiences, cataloguers and archivists. But, since the 1990s, few historians have engaged with his historiographical approaches.^142^ Nor has there been much about other of his themes that resonate today: His arguments about the role of war in shaping the development of medical and public health films,^143^ or how history should relate to efforts to reform film or other visual practices.

Such tendencies – to avoid critical engagement with his broader historiographical approaches and arguments – suggest a final paradox of Nichtenhauser. Despite his recent resurrection, his arguments and approaches remain marginalized, subordinated to his discussion about this or that innovation, person, or event. He is recognized as an important source of information, even an authority, but rarely as a scholar whose larger arguments should be engaged critically.

## Footnotes

1 Adolf Nichtenhauser, *A History of Motion Pictures in Medicine* (unpublished manuscript, circa 1950), Archives and Modern Manuscripts Collection, National Library of Medicine, MS C 380. (hereafter cited as ‘Nichtenhauser, *History*’). The book is in 4 volumes identified as Vol I, Vol. II, Vol. III, and Vol. IV. Please note that Nichtenhauser himself does not label the first volume as Volume I and that I have included the prelims in this first volume. Work derived from this book is published in Adolf Nichtenhauser , ‘A Half-Century of Motion Pictures in Neurology, Psychiatry, Psychology, and Mental Health Education’, in Adolf Nichtenhauser , Marie L. Coleman , and David S. Ruhe (eds.), Films in Psychiatry, Psychology & Mental Health (New York: New York Health Education Council, 1953), 44–54 . Adolf Nichtenhauser , ‘A Historical Perspective on Short Films in Medical Education’, Journal of Medical Education, 28, 2 (February 1953), 53–61 13023307.

2 Adolf Nichtenhauser Medical Film Materials, Archives and Modern Manuscripts Collection, National Library of Medicine, MS C 277. (Hereafter cited as ‘Nichtenhauser Collection, NLM’.) David Cantor, ‘Adolf Nichtenhauser: Documenting the History of Movies and Medicine’, *Circulating Now* (From the Historical Collections of the National Library of Medicine), 3 March 2022, https://circulatingnow.nlm.nih.gov/2022/03/03/adolf-nichtenhauser-documenting-the-history-of-movies-and-medicine/ (accessed 8 December 2025)

3
https://findingaids.nlm.nih.gov/repositories/ammp/resources/nichtenhauser277; https://findingaids.nlm.nih.gov/repositories/ammp/resources/nichtenhauser380. (Both accessed 8 December 2025)

4 Books that make use of the manuscript include the following: Christian Bonah , David Cantor , and Anja Laukötter (eds.), Health Education Films in the Twentieth Century (Rochester: Rochester University Press, 2018). Leslie J. Reagan , Nancy Tomes , and Paula A. Treichler (eds.), Medicine’s Moving Pictures: Medicine, Health, and Bodies (Rochester: Rochester University Press, 2018). Scott Curtis , The Shape of Spectatorship: Art, Science, and Early Cinema in Germany (New York: Columbia University Press, 2015). Martin S. Pernick , The Black Stork: Eugenics and the Death of ‘Defective’ Babies in American Medicine and Motion Pictures Since 1915 (New York and Oxford: Oxford University Press, 1996). Lisa Cartwright , Screening the Body: Tracing Medicine’s Visual Culture (Minneapolis and London: University of Minneapolis Press, 1995).

5 Christian Bonah, David Cantor, and Anja Laukötter, ‘Introduction’, in Bonah, Cantor, and Laukötter, *op. cit.* (note 4), 19–22.

6 The missing sections are two chapters on post-Second World War developments: ‘Health and Related Films since the End of World War II’ and ‘Medical Films from the End of World War II to the Present’. It may be that they were cut from the volume, as the outline of the following chapter ‘The Lesson of the Past and the Task of the Present’, begins on the page vacated by the earlier of the two missing chapters in Vol. IV, 282.

7 On the complex identities of Jews in Moravia, see for example, Marsha L. Rozenblit , ‘Jews, German Culture, and the Dilemma of National Identity: The Case of Moravia, 1848–1938’, Jewish Social Studies, 20 (2013), 77–120 . Jan Láíček , Arnošt Frischer and the Jewish Politics of Early 20th-Century Europe (New York: Bloomsbury Academic, 2017). See also, Kateřina Čapková , Czechs, Germans, Jews? National Identity and the Jews of Bohemia (New York and Oxford: Berghahn Books, 2012).

8 Walter Mentzel, ‘Adolf Nichtenhauser – Ein in Österreich vergessener Pionier des medizinischen Films und Autor des unveröffentlichten Manuskripts: “A History of Motion Pictures in Medicine”’, 21 September 2017 at https://ub-blog.meduniwien.ac.at/blog/?p=28159 (accessed 6 September 2024). See also, for background, Mečislav Borák , ‘Internace cestujících z lodi Alsina v západní Africe v roce 1941 a jejich další osudy’, Ostrava. Příspěvky k dějinám a současnosti Ostravy a Ostravska, 30 (2016), 37–65 . A short article by Katrin Pilz – ‘Zwischen Medizin und Filmleinwand: Adolf “Optikus” Nichtenhauser – Eine Exilgeschichte,’ *Biographie des Monats*, September 2025, https://www.oeaw.ac.at/acdh/oebl/biographien-des-monats/2025/september (accessed 8 December 2025) – appeared after this article went to press.

9 For another study that shows how films created a useful past see Dimitrios Latsis , How the Movies Got a Past: A Historiography of American Cinema, 1894–1930 (New York: Oxford University Press, 2023).

10 More generally on the reasons for the migration of Austrian Jews before the Anschluss see Gabriele Anderl , ‘“Departure from a Sinking World”: Jewish Emigration from Austria between 1933 and 1938’, The Leo Baeck Institute Year Book, 66 (2022), 58–78 . On Jewish experiences in Vienna see George E. Berkley , Vienna and Its Jews: The Tragedy of Success, 1890s–1980s (Cambridge, MA and Lanham: Abt Books/Madison Books, 1988). Steven Beller , Vienna and the Jews, 1867–1938: A Cultural History (Cambridge and New York: Cambridge University Press, 1989). Bruce F. Pauley , From Prejudice to Persecution: A History of Austrian Anti-Semitism (Chapel Hill: University of North Carolina Press, 1992).

11 Borák, *op. cit.* (note 8), 37–65.

12 Most accounts list Vienna as Nichtenhauser’s place of birth. An exception is the Matriculation album from the Rheinische-Friedrich-Wilhelms Universität Bonn (signature: AB-18 1918-1922) which lists his birthplace as Mährisch Ostrau, Mähren, Tscheslowakei.

13 Notes that Mečislav Borák took for his article, Borák, *op. cit.* (note 8), 37–65. Thanks to his son, Matouš Borák, for emailing the notes to me 15 March 2023.

14 Adolf Nichtenhauser document stamped 20 November 1946, File ‘Adolf Nichtenhauser’, Record Group 299 Records of the Foreign Claims Settlement Commission of the United States 1915–1999, Series Correspondence Relating to Czech Claims 1958–1983, National Archives (hereafter NARA), College Park, Maryland. Thanks to Oliver Gaycken for providing this documentation.

15 Karel Nichtenhauser, domovské právo, 7 April 1931, Card file of home citizens of Moravská Ostrava – Karel Nichtenhauser, card no. 740, Magistrát města Ostravy, Archiv města Ostravy, Archivní oddělení (AMO) [Municipality of Ostrava, Archive of the City of Ostrava, Archives Department] Spalova 19, 702 19 Ostrava-Privoz.

16 The family lived at the same address in 1910 and 1921; 1364/20 Nová Street (called Českých legií in 1921).
https://badatelna.ostrava.cz/vademecum/permalink?xid=6CD30FF0A3C411E9B75E000C29D8B843&scan=9823174a8c69488a90e800e469d3822d (1910 Census) (accessed 9 December 2025)
https://badatelna.ostrava.cz/vademecum/permalink?xid=B204A0AE240911EBB794000C29D8B843&scan=3072c58abc3d43b7870642319c400458 (1921 Census) (accessed 9 December 2025)

17
*Kur- und Bade-Zeitung den östenreichischen Riviera*, Nr. 20 Abbazia (19 January 1907), 11. *Kurliste Bad Ischl*, No. 19 (4 August 1932). *Kurliste Bad Ischl*, No. 22 (12 August 1932). *Fremden-Liste de Kurstadt Gmunden, Oberosterreich*, No. 4 (6 August 1932), 2. *Gmunder Kurliste*, No 14, Season 49, (20 July 1910), and possibly *Karlesbeder Kurliste*, No 269 (21 June 1906). *Alphabetisch geordnetes Namensverzeichnis. Kurgäste Karlsbad’s*, No. 70 (11 August 1905).

18
https://badatelna.ostrava.cz/vademecum/permalink?xid=262a90fa00414db8b23902e3b6c4c5ed&scan=14f95258581a4e15b35f02e6c2225625 (accessed 20 June 2024)

19 German Real Gymnasium (Realgymnasium), report cards of Adolf and Karel Nichtenhauser, GR VIII – 41, school year 1917–1918, year 5a; GR VIII – 43, school year 1919–1920, year 1a, AMO. See also Borák, *op. cit.* (note 8), 37–65. Borák claims he obtained this qualification in 1920, but this likely refers to his brother, Karel, who obtained his certificate in that year. For a history of this school to 1914 see Petr Kadlec , ‘K historii německé reálky v Moravské Ostravě do roku 1914’, Vlastivědný věstník moravský, 66 (2014), 372–80. And on Jewish schools in the same city, see Marsha L. Rozenblit , ‘Creating Jewish Space: German-Jewish Schools in Moravia’, Austrian History Yearbook, 44 (2013), 108–47.

20
**Friedrich-Wilhelms-Universität zu Berlin (30/10/1920–08/03/21)** (1 semester) Subject: Naturwissenschaften (natural sciences), Faculty of Philosophy. Thanks to Heather Forster (Universitätsarchiv der Humboldt-Universität zu Berlin) for her email, 17 March 2023.
**Rheinische-Friedrich-Wilhelms Universität Bonn (1921)** Summer Semester, matriculated 27 April 1921 in philosophy. Student directories – (signature: PVSV 1921), Matriculation Album (signature: AB-18 1918–1922). Thanks to Anika Zimmerman of the Rheinische-Friedrich-Wilhelms Universität Bonn Archive for her email, 13 March 2023)
**Universität Heidelberg.** (**Winter Semester 1921–22; Summer Semester 1922)** Courses included ones on German literature, German history, the German Renaissance painter Mathias Grünewald, Psychology, German dramas, Archaeology, Art history, Latin, zoology, Chemistry, and human evolutionary history. Thanks to Sabrina Zinke, Deputy Head of Archives, Heidelberg University Archives, for providing scans of Nichtenhauser’s records.
**Friedrich-Wilhelms-Universität zu Berlin (02 May 1923–30 August 1924)** (3 semesters) Subject: Zoologie at the Faculties of Philosophy and Medicine. Thanks to Heather Forster (Universitätsarchiv der Humboldt-Universität zu Berlin) for her email, 17 March 2023.
**Universität Wien (1924–31)**: Nichtenhauser began as a student of medicine in the winter semester 1924–25 and graduated with a Doctor medinae universalis on 12 December 1931. Thanks to Thomas Maisel’s (Archiv der Universität Wien) email to David Cantor, 7 March 2023.

21 1931–3: Internship, Vienna General Hospital, Rotating internship. 1933–4: Assistant physician, Municipal Hospital for Chronic Diseases, Vienna; Worked in Departments of Tuberculosis and Surgery. 1934–6: Attending Physician, Vienna Hospitals; Medicine University Clinic, Gynaecological University Clinic, St. Anna Children’s Hospital, Workmen’s Insurance Clinic. 1936–7: Staff physician, Cottage Sanatorium, Vienna; Diagnosis and treatment of internal diseases. See Adolf Nichtenhauser, ‘Curriculum Vitae’, April 1952, Nichtenhauser Collection, Folder, ‘Nichtenhauser, Adolf – biographical’.

22 Nichtenhauser helped translate into German the first half of the symposium, Glandular Physiology and Therapy, published by the American Medical Association, and prepared German abstracts from American periodicals for *Ars Medici* (Vienna). Adolf Nichtenhauser and Kurt Stern (trans.), Die Drüsen Mit Innerer Sekretion: Ihre Physiologische und Therapeutische Bedeutung: Ein Sammelwerk über die Gesamte Endokrinologie; Autorisierte Übersetzung und Erweiterung (Wien: Aesculap-Verlag, 1937). See Nichtenhauser, ‘Curriculum Vitae’, *Ibid.*

23 Adolf Nichtenhauser, ‘Personal History’, c. February 1945, Box 15, Folder 18, ‘Nichtenhauser, Adolf, 1945’, Wesley H. Greene Papers, Special Collections Research Center, University of Chicago Library.

24 According to his CV, Nichtenhauser took two courses in motion picture technology: 35 mm, Vienna School of Engineering (1926–7); 16 mm, Technological Museum, Vienna (1937). Nichtenhauser, ‘Curriculum Vitae’, *op. cit.* (note 21). Nichtenhauser, ‘Personal History’, *ibid.* However, he appears in none of the surviving records of either school.
**35-mm course**: The ‘Vienna School of Engineering’ is likely the engineering school, then part of the Technische Hochschule, but Nichtenhauser does not appear in the latter’s records either as a regular or an extraordinary student. It is possible that he attended as a guest student, which could explain his absence from the records. Thanks to Mirko Herzog (Technisches Museum Wien, archiv) and Alexandra Wieser (Technische Universität Wien, Archiv). A lecture titled Technische und wissenschaftliche Kinematographie (Technical and Scientific Cinematography) started at the Technische Hochschule in 1924–5. The course description for 1926–7, the year Nichtenhauser may have attended is at ‘Technische und wissenschaftliche Kinematographie’, *Vorlesungsverzeichnis, Studienpläne und Personalstand der Technischen Hochschule in Wien für das Studienjahr 1926/27* (Wien: Verlag der Technischen Hochschule), 37.
**16-mm course**: The ‘Technological Museum, Vienna’ could refer to either the Technisches Museum Wien (TMW – also called the Technisches Museum für Industrie und Gewerbe) or the ‘Technologisches Gewerbemuseum’ (TGM). The TMW archivist Mirko Herzog did not find any announcement of courses in 1937, though there were some lectures on the subject, and Nichtenhauser does not appear in their records. As regards the TGM the librarian Carina Gröger could not find any teaching course on film technology for the years 1935–6 and 1936–7, the last year of publication of the annual reports until 1945–6. Nichtenhauser does not appear in their records.

25 Nichtenhauser, *op. cit.* (note 23). For a report on the film which does not seem to have a title, see ‘Das Pfingstjugendtreffen der Kaufmännischen Angestellten im Schmalfilm’, *Das Kleine Blatt* (25 December 1930), 15.

26 Nichtenhauser, *Ibid.*

27

Stefanie Oswalt , Die Weltbühne: Zur Tradition und Kontinuität Demokratischer Publizistik: Dokumentation der Tagung ‘Wieder gilt: Der Feind steht Rechts!’ (St. Ingbert: Röhrig, 2003). Hans-Albert Walter . Deutsche Exilliteratur 1933–1950, Band 4 (Stuttgart: J.B. Metzler Verlag, 1978). 23–71. Alexander Gallus , Heimat ‘Weltbühne’: Eine Intellektuellengeschichte Im 20. Jahrhundert. Hamburger Beiträge zur Sozial- und Zeitgeschichte, Band 50 (Göttingen: Wallstein Verlag, 2012).

28 Nichtenhauser, *op. cit.* (note 23).

29 Nichtenhauser, ‘Curriculum Vitae’, *op. cit.* (note 21).

30 Adolf Nichtenhauser, ‘Was Wird aus dem deutschen Film?’ *Wiener Weltbühne* (30 March 1933), 398–401. Opticus (a pseudonym adopted by Nichtenhauser in some publications in the 1930s, hereafter cited as Adolf Nichtenhauser), ‘Der Film im neuen Österreich’, *Zeitschau*, 14 (December 1934), 25–7 and No. 15, 23–5. Adolf Nichtenhauser, ‘Die völkerverbindenden Aufgaben der deutschen Filmkunst’, *Der Bühnenkünstler*, Zürich (April 1936), 16–19. Nichtenhauser’s CV also lists as one of his own articles an article by Ernst Wolfe (another pseudonym?) ‘Ein Filmprophet des Dritten Reichs’, *Die Neue Weltbühne* (25 May 1933), 657–58. In this review, the writer (Wolfe? Nichtenhauser?) saw Fritz Lang’s *The Testament of Dr. Mabuse* (1933) as forewarning of the dangers of fascism. In addition, many of his publications in *Das Kleine Blatt* and the *Allgemeines Illustriertes Wochenendblatt* concerned the effects of the political situation on films. Nichtenhauser, ‘Curriculum Vitae’, *op. cit.* (note 21).

31 On the ban, see Prof. Egon Ranzi (head of the I. Chirurgische Universitätsklinik) to Dekanat der medizinischen Fakultät der Universität (Dean of the Faculty of Medicine), 15 November 1935, File Ref. Code: Med. Dekanat Gesch. -Zl. 956 aus 1935/36, Archiv der Universität Wien. Thanks to Thomas Maisel for this information. The records on the accusations of anti-government propaganda abroad are in the Bundesministerium für soziale Verwaltung, Sektion Volksgesundheit (Federal Ministry of Social Affairs, Division of Public Health), Österreichisches Staatsarchiv. These are entries in the minute books, and a search by Isabella Riedel of the Österreichisches Staatsarchiv found none of the actual files when going through the corresponding file boxes, perhaps because all the file numbers concerning Adolf Nichtenhauser were marked as confidential (unter Verschluss) in the books. They were probably kept separately and at some point, may have gotten lost. The references in the minute books are as follows: BMfsV VG Protokoll 3.774 ex 34; BMfsV VG Protokoll 57.422 ex 33; BMfsV VG Protokoll 82.747 ex 33; and BMfsV VG Protokoll 84.902 ex 33. Thanks to Isabella and also to Walter Mentzel for alerting me to these records. It is unclear to what anti-government propaganda the accusation might refer. Likely, it referred to his many publications in international journals cited elsewhere in this paper. Nichtenhauser was also a regular letter writer with foreign correspondents, for example, see Adolf Nichtenhauser letter, 22 March 1933, ‘Adolf Nichtenhauser, 1932 oktober-1933 oktober’, Archief van de redactie van het tijdschrift Filmliga (UBA178), Item Identifier: Hs. XVI A 9:241. Allard Pierson Repository (Oude Turfmarkt 127–129, Amsterdam 1012 GC Nederland). Correspondence between Adolf Nichtenhauser and Dr. W. W. Charters 7 May 1934, 28 May 1934, 19 September 1934, 8 October 1934, 28 October 1934, 13 November 1934, and 22 January 1935, Werrett Wallace Charters Papers. RG 40.117, File ‘Nichtenhauser, Adolf 1934-5’, Box UA.RG.40.117.0012, Folder 36, University Archives (The Ohio State University, 132 Book Depository, 2700 Kenny Road, Columbus, OH 43210).

32 See note 21. Hostility towards Jewish doctors and students, particularly at the University of Vienna, is documented for the interwar period in Birgit Nemec and Klaus Taschwer , ‘Terror gegen Tandler. Kontext und Chronik der antisemitischen Attacken am I. Anatomischen Institut der Universität Wien, 1910 bis 1933’, in Oliver Rathkolb (ed.), Der lange Schatten des Antisemitismus. Kritische Auseinandersetzungen mit der Geschichte der Universität Wien im 19 und 20. Jahrhundert (Göttingen: Vienna University Press, 2013), 147–172 .

33 Adolf Nichtenhauser, ‘Der Aufbau des medizinischen Filmwesens’, *Wiener Medizinischen Wochenschrift*, 28 (7 July 1934), 784–787. The publication of an article in this journal suggests that despite his political problems he retained some good connections with people in the Medical School. It could be difficult to publish in the *Wiener Medizinischen Wochenschrift* without such connections.

34 Adolf Nichtenhauser, ‘Kulturarbeit am Film’, *Der Bücherwurm*, Vienna (October 1933), 2–4. Adolf Nichtenhauser, ‘Der Schmalfilm’, *Frankfurter Zeitung* (25 September 1932), 10. Adolf Nichtenhauser, ‘Der Aufbau der Kulturarbeit im Film’, *Internationale Lehrfilmschau* (April 1933), 259–89. English translation: Adolf Nichtenhauser, ‘Progress in the Evolution of Cultural Films’, *International Review of Educational Cinematography* (April 1933), 243–269.

35 See, for example, Katrin Pilz , ‘Aufklärungs-, Lehr-, oder Bildungsfilm? Der Wiener Hygienefilm der 1920er-Jahre’, Spurensuche: Zeitschrift für Geschichte der Erwachsenenbildung und Wissenschaftspopularisierung, 27 (2018), 45–59 . Katrin Pilz , ‘Re-Edited Medical Films in Vienna: Vom “physiologischen Theater” zum chirurgischen Filmset und “orthopädischen Filmzirkus”’, in Delia González de Reufels , Rasmus Greiner , Stefano Odorico and Winfried Pauleit (eds.), Film als Forschungsmethode. Produktion – Geschichte – Perspektiven (Bremen: Bertz + Fischer, 2018), 91–100. http://www.bertz-fischer.de/product_info.php?products_id=524 . For a database of historical Austrian educational films and associated materials, see https://www.lehrfilmpraktiken.at/Front/Index/lang/en_US. At the time of writing, this only includes one entry by Nichtenhauser: Nichtenhauser, *op. cit.* (note 33), which is a source for this paragraph.

36 On medical film as replacement for live experiments in France, see Christian Bonah and Joel Danet , ‘“See for Yourself:” Autopsy Film as Audio-Visual Mediation of Learning Experiences, c.1928–1962’, in John Nott and Anna Harris (eds.), Making Sense of Medicine: Material Culture and the Reproduction of Medical Knowledge (Bristol: Intellect Books, 2022), 125–46.

37 Prof. Egon Ranzi (head of the I. Chirurgische Universitätsklinik) to Dekanat der medizinischen Fakultät der Universität (Dean of the Faculty of Medicine), 15 November 1935, File Ref. Code: Med. Dekanat Gesch. -Zl. 956 aus 1935/36, Archiv der Universität Wien. Thomas Maisel (Archiv der Universität Wien) email to David Cantor, 7 March 2023.

38 Folder ‘Correspondence, 1937–1941’, Nichtenhauser Collection, NLM.

39 Folders ‘Correspondence, 1933–1936’ and ‘Correspondence, 1937–1941’, Nichtenhauser Collection, NLM. His articles for the *Motion Picture Herald* include Adolf Lichtenhauser [*sic*], ‘American Product Maintains Lead in Austria Despite Kontingent [*sic*] Law’, *Motion Picture Herald*, 123, 4 (25 April 1936), 61. Adolph [*sic*] Nichtenhauser, ‘New Society in Vienna to Show Top Foreign Films’, *Motion Picture Herald*, 123, 12, (20 June 1936), 64. Adolf Nichtenhauser , ‘U.S. Film Showing Set First Time at Salzburg Festivals’, Motion Picture Herald, 124, 3 (18 July 1936), 78 ; Adolf Nichtenhauser , ‘Films at Salzburg Festival Win Press, Public Approval’, Motion Picture Herald, 124, 9 (29 August 1936), 41 . Adolf Nichtenhauser , ‘Licensing System Limits Vienna Theatre Growth’, Motion Picture Herald, 128, 11 (11 September 1937), 85–86 . See also Adolf Nichtenhauser , ‘An International Catalogue of Important Films Urged,’ The Film Daily, 64, 1 (20 October 1933), 3 .

40 Kenneth Macgowan to American Consul, Paris September 10, 1938. Folder ‘Correspondence, 1937–1941’, Nichtenhauser Collection, NLM. Lisa Gerlach ‘“I think he will be able to adjust”. Letters of Recommendation Accompanying Jewish Refugees in Transit during National Socialism’, The Leo Baeck Institute Year Book, 69, 1 (2024), 14–31 .

41 List or Manifest of Alien Passengers for the United States Immigrant Inspector at Port of Arrival, New York, 8 February 1939, S/S ‘Paris’, Passengers sailing from Havre, France, 1 February 1939, *Passenger and Crew Lists of Vessels Arriving at New York, New York, 1897–1957*; Microfilm Serial or NAID: *T715*; RG Title: *Records of the Immigration and Naturalization Service, 1787–2004*; RG: *85*, NARA, Washington, DC.

42 On intellectual immigration to the United States, see Laurel Leff , Well Worth Saving: American Universities’ Life-and-Death Decisions on Refugees from Nazi Germany (New Haven: Yale University Press, 2019). Donald Fleming and Bernard Bailyn (eds.), The Intellectual Migration: Europe and America, 1930–1960 (Cambridge: Belknap Press of Harvard University Press, 1969). Jarrell C. Jackman and Carla M. Borden (eds.), The Muses Flee Hitler: Cultural Transfer and Adaptation, 1930-1945 (Washington, DC: Smithsonian Institution Press, 1983). Laura Fermi , Illustrious Immigrants: The Intellectual Migration from Europe 1930–41, 2nd edn (Chicago and London: University of Chicago Press, 1971). Anthony Heilbut , Exiled in Paradise: German Refugee Artists and Intellectuals in America, from the 1930s to the Present (New York: Viking Press, 1983). Peter Kielmansegg , Horst Mewes , and Elisabeth Glaser-Schmidt , Hannah Arendt and Leo Strauss: German Émigrés and American Political Thought after World War II (Washington, DC, and Cambridge: Cambridge University Press, 1995).

43 Adolf Nichtenhauser to Alan Gregg, 25 March 1943, Rockefeller Foundation Archives (Rockefeller Archive Center, 15 Dayton Avenue, Sleepy Hollow, New York 10591), RG 1.1, Series 200, Box 70, Folder 842. Foundation Archives, Folder: American Film Center – Medical Films 1943, RG 1.1, Series 200, Box 70, Folder 842. According to the ship’s manifest Nichtenhauser paid for Bedřich’s passage from Casablanca to New York. List or Manifest of Alien Passengers for the United States Immigrant Inspector at Port of Arrival, New York, 9 August 1941, S. S. Nyassa, Passengers sailing from Casablanca 26 July 1941, *Passenger and Crew Lists of* Vessels *Arriving at New York, New York, 1897–1957*; Microfilm Serial or NAID: *T715*; RG Title: *Records of the Immigration and Naturalization Service, 1787–2004;* RG: *85*, NARA, Washington, DC.

44 On antisemitism in the universities, see Leff , *Well Worth Saving*, *op. cit.* (note 42), Chapter 6. Also, Leonard Dinnerstein on challenges to antisemitism, ‘Anti-Semitism Exposed and Attacked, 1945–1950’, American Jewish History, 71 (1981), 134–49.

45 Robert E. Park, ‘Human Migration and the Marginal Man’, *American Journal of Sociology*, 33, 6 (1928), 881–93, at 892.

46 Adolf Nichtenhauser, Declaration of Intention, June 22, 1939, NAI Title: Declarations of Intention for Citizenship, 1/19/1842–10/29/1959; NAI Number: 4713410; Record Group Title: Records of District Courts of the United States, 1685-2009; Record Group Number: 21, NARA, Philadelphia.

47 Margaret survived Adolf’s death, marrying a Hugo Gruenbaum in 1959. For her death see: Margaret H. Gruenbaum, *Social Security Death Index, Master File*, Social Security Administration, Washington D.C. A declaration of intention (6 November 1939) for a Margaret Regina Hirsch with the same birth date is at NAI Title: *Declarations of Intention for Citizenship, 1/19/1842 - 10/29/1959*; NAI Number: 4713410; Record Group Title: Records of District Courts of the United States, 1685-2009; Record Group Number: 21, NARA, Philadelphia. Thanks to Michael Trotter for these leads.

48 Among his addresses were as follows: 1939: 29 W. 71 St, NY. 1942: 105 W. 74th St, NY.

49 Manifest of Alien Passengers S. S. Nyassa, *op. cit.* (note 43). ‘ZU Washington 1921–50’, File, ‘Socialni-Uprchlici v Africe-lod Alsina, No 2174.41 ze dne 29.5.1941,’ Archiv Ministerstva zahraničních věcí (AMZV – Archive of the Ministry of Foreign Affairs, Czech Republic). Thanks to Tomas Kluson for his help in locating these records. For more context on Bedřich’s route to the United States, see Borák, *op. cit.* (note 8), 37–65. Bedřich seems to have come direct to the United States from Casablanca, where many of those who escaped Europe on the SS Alsina were stranded. Other refugees transited through Curaçao, but they do not seem to have included Bedřich; see the file ‘Archief van het Gouvernement van Curaçao, (1851) 1938–1951 (1957),’ ‘Archief Oorlogsjargen’, (Archives on the war years) Inventory Number 1364, File name, ‘Van (onder meer Joodse) vluchtelingen aan boord van S.S. Cabo de Homos en S.S. Cabo de Buena Esperanza, 1941–1946,’ Nationaal Archief Curaçao (Scharlooweg 77–79, Willemstad, Curaçao). My thanks to Glendalin William for her help in locating this file during my visit to the archives 20 July 2023. For more information on Curaçao and the Cabo de Hornos, see Ron Gomes Casseras and Jos Rozenburg , ‘Cabo de Hornos’, Lantèrnu 26 (2022), publication of the Nationaal Archief Curaçao. On rescuing and caring for his brother see Adolf Nichtenhauser to Alan Gregg, 25 March 1943, Rockefeller Foundation Archives, RG 1.1, Series 200, Box 70, Folder 842.

50 Manifest of Alien Passengers, S/S ‘Paris’, *op. cit.* (note 41). In Germany, Dora was associated with the circle around liberal politician, Friedrich Naumann. A pacifist, she emigrated to the United States in 1935, and in the 1940s, she worked with the Emergency Rescue Committee, helping intellectuals, artists and socialists flee from Nazi-occupied Europe. Likely she helped her cousin Adolf too, and perhaps Bedřich. On her speciality as a philologist see Dora Nichtenhauser, Naturalization Oath of Allegiance, 4 July 1944, NAI Title*: Index to Petitions for Naturalizations Filed in Federal, State, and Local Courts in New York City, 1792–1906*; NAI Number: 5700802; Record Group Title: Records of District Courts of the United States, 1685–2009; Record Group Number: 21, NARA, Washington, DC. For her thesis, see Dora Nichtenhauser, ‘Rückbildungen im Neuhochdeutschen’ (Doctoral thesis: Albert-Ludwigs-Universität zu Freiburg i. B, 1920). On Dora’s involvement in helping Jewish refugees, see Anne Klein , Flüchtlingspolitik und Flüchtlingshilfe 1940–1942: Varian Fry und die Komitees zur Rettung politisch Verfolgter in New York und Marseille (Berlin: Metropol, 2007), 163 .

51 Adolf Nichtenhauser, Draft registration card, 15 February 1942, *WWII Draft Registration Cards For New York City, 10/16/1940*-*03/31/1947*; Record Group: *Records of the Selective Service* System, *147*L., NARA, St. Louis, Missouri. According to her naturalization records, Lola – also known as Charlotte Lola Nichtenhauser – had arrived in the United States in 1923, before either Dora or Adolf. Her naturalization document lists her occupation as nurse-teacher. Charlotte Lola Nichtenhauser, Naturalization Oath of Allegiance, 3 October 1938, Petition No. 309422–Petition No. 309725, *Petitions for Naturalization from the U.S. District Court for the Southern District of New York, 1897–1944*; Series: M1972; Roll: 1139, NARA, Washington, DC. Her relationship to Dora, then still living in Germany (she is listed at Schloss Sachsenburg SA, Frankenberg) is noted in a shipping manifest from 1928. List or Manifest of Alien Passengers for the United States Immigrant Inspector at Port of Arrival, New York, 9 August 1941, S. S. Muenchen. Passengers sailing from Bremen, 25 October 1928, *Passenger and Crew Lists of Vessels Arriving at New York, New York, 1897–1957*; Microfilm Serial or NAID: *T715*; RG Title: *Records of the Immigration and Naturalization Service, 1787–2004*, RG: 85, NARA, Washington, DC.

52 The dates of Hermann and Ida’s death come from the website Memory of Treblinka – https://memoryoftreblinka.org/ (accessed 24 September 2024) – which also details their journey from Moravská Ostrava/Mährisch Ostrau.

53 Joseph Savoretti by T. B. Shoemaker to Honorable J., W. Pehle, 1 December 1944, Records of the War Refugee Board, 1944–1945, Series 1: General Correspondence, Box 13, Folder, ‘Issuance and Reissuance of US Visas December 1–31, 1944 (Folder 3 of 3)’, Franklin D. Roosevelt Presidential Library & Museum, http://www.fdrlibrary.marist.edu/_resources/images/wrb/wrb0278.pdf (accessed 14 February 2023).

54 Adolf Nichtenhauser, ‘The Private Lives of Walt Disney’s Figures’, 4 April 1939. Rejection letter: Ellen Kennedy to Paul Kohner May 23, 1939. Both in Folder: Correspondence, 1937–1941, Nichtenhauser Collection, NLM.

55 Note by John Grierson scrawled on Wesley Greene memo to John Grierson, 17 February 1945, Box 15, Folder 18, ‘Nichtenhauser, Adolf, 1945’, Wesley H. Greene Papers, Special Collections Research Center, University of Chicago Library.

56 Alan Gregg to Adolf Nichtenhauser, 8 March 1945, Rockefeller Foundation Archives, RG 1.1, Series 200, Box 70, Folder 843.

57 ‘Health Films Sponsored. Section Is Established at the American Center Here’, *New York Times* (9 October 1941), 26. In 1944, the Foundation made a further grant of $8,600 to the Center for support of this program for an additional two-year period. *The Rockefeller Foundation Annual Report 1944* (New York: The Foundation), 145–6. Nichtenhauser, ‘Curriculum Vitae’, *op. cit.* (note 21).

58 On the AFC, see Tino Balio , Grand Design: Hollywood as a Modern Business Enterprise, 1930–1939 (Berkeley: University of California Press, 1993), 374 and especially Victoria Cain , ‘“An Indirect Influence Upon Industry”: Rockefeller Philanthropies and the Development of Educational Film in the United States, 1935–1953’, in Devin Orgeron , Marsha Orgeron , and Don Streible (eds.), Learning with the Lights Off: Educational Film in the United States (Oxford: Oxford University Press 2012), 230–48, esp. 243–47.

59 From AG’s diary, 3 January 1945, Donald Slesinger – phone, Rockefeller Foundation Archives, RG 1.1, Series 200, Box 70, Folder 843. On Slesinger, see “Donald Slesinger, a Psychologist And Ex-Law School Dean, Is Dead,” *New York Times* (14 October 1977), 28.

60 Memo, HRL [Hugh R. Leavell], Monday 17 December 1945, Rockefeller Foundation Archives, RG 1.1, Series 200, Box 70, Folder 843.

61 From AG’s diary, 10 January 1945, Adolf Nichtenhauser, Rockefeller Foundation Archives, RG 1.1, Series 200, Box 70, Folder 843. For more context on problems at the AFC, see Cain, *op. cit.* (note 58), 243–47.

62 Memo, HRL *op. cit.* (note 60). See also WW Peter to Alan Gregg, 8 January 1945, Rockefeller Foundation Archives, RG 1.1, Series 200, Box 70, Folder 843.

63 A.N. ‘Aide Memoire. Conversation with Dr. Schultz, morning of April 13, 1948’, File, ‘Schultz, Robert’, Nichtenhauser Collection, NLM.

64 [Adolf Nichtenhauser], ‘Aide Memoire. Conversation with Dr. Schultz, April 13,1948, relative to putting my name on the long Foundation statement’, File, ‘Schultz, Robert’, Nichtenhauser Collection, NLM.

65
*Ibid.*

66 For Nichtenhauser’s proposal for a critical survey of the present state of motion pictures in education, science, and cultural life, see Adolph Nichtenhauser to Capt. R.V. Schultz, 2 November 1946, Folder, ‘Navy Med. Film Project, 1945–1947’, Nichtenhauser Collection, NLM. For a draft of the request for his appointment, see ‘Memorandum to Rear Admiral M. D. Willcutts (MC), USN: Subj: A study of the role of the film in medical education – Request for’, 6 January 1947, Folder, ‘Navy Med. Film Project, 1945–1947’, Nichtenhauser Collection, NLM. As this file demonstrates this proposal was in the context of Nichtenhauser developing proposals for the reorganization of medical and health film production, research, and distribution, including the creation of a Medical Film Institute. See also the file ‘Official papers’, Nichtenhauser Collection, NLM. The contract was later transferred to the Office of Naval Research and broadened to encompass the history of films in medicine. The Film Library of the Museum of Modern Art (MOMA) became the grant administrator, while Nichtenhauser continued to broaden and polish the draft. Series IV, Folder 247, ‘Film Library: Nichtenhauser Report’, René d’Harnoncourt Papers, The Museum of Modern Art Archives (11 West 53 Street, New York, NY 10019-5497). See also the files ‘Office of Naval Research – (Museum of Modern Art)’, and ‘Office of Naval Research – Miscellaneous’, Nichtenhauser Collection, NLM.

67 Nichtenhauser, *History*, *op. cit.* (note 1), Vol. I, pp. II-III, and VIII.

68 Nichtenhauser, *History*, *op. cit.* (note 1), Vol. I, p.III. For another historian joining Nichtenhauser as a prisoner, see Oliver Gaycken , Devices of Curiosity: Early Cinema and Popular Science (New York: Oxford University Press, 2015), ix.

69 Nichtenhauser, *History*, *op. cit.* (note 1), Vol. I, p.III.

70 Robert V. Schultz to Adolf Nichtenhauser 7 January 1952, and Adolf Nichtenhauser to Robert V. Schultz, 18 January 1952, Folder, ‘Schultz, Robert’, Nichtenhauser Collection, NLM.

71 Parts of this section are derived from Bonah, Cantor, and Laukötter, *op. cit.* (note 5), 19–22.

72
*Ibid.*, 20.

73
*Ibid.*

74 Nichtenhauser, *History*, *op. cit.* (note 1), Vol. IV. Bonah, Cantor, and Laukötter, *Ibid.*, 20–21.

75 On commercial neglect: Nichtenhauser, *History*, *Ibid.*, Vol. I, 105–6. State involvement Vol. I, 109.

76
*Ibid.*, Vol. II, 65.

77 On US interest in medical film during the First World War, see *Ibid.*, Vol. I, 131, 135–141, and 159–225; Vol. II, 65.

78 Pathé-Rural: *Ibid.*, Vol. III, 54.

79
*Ibid.*, 137–138 and 281 (HF 1).

80
*Ibid.*, 295–96 (HF15-16). *March of Time*: *Ibid.*, 287–88 (HF6-7). Raymond Fielding , The March of Time, 1935–1951 (New York: Oxford University Press, 1978). Gracia Ramírez , ‘
*The March of Time* in Britain and the International History of Documentary Film,’ Film History: An International Journal, 32 (2020), 1–27 . Gracia Ramírez , ‘
*The March of Time*: News Documentaries and the Dramaturgy of History,’ Film & History: An Interdisciplinary Journal, 54 (2024), 39–58 .

81 VD films and commerce: Nichtenhauser, *Ibid.*, Vol. II, 81–2. On later developments regarding sex, VD, and film, see Vol. III, 282–86 (HF2-6) and 314–15 (HF34-5).

82 On distrust of UFA as a commercial organization, see *Ibid.*, Vol. II, 18; distrust of commercially sponsored films Vol. II, 125; how commercial interests frustrated international developments Vol. IV, 4.

83
*Ibid.*, Vol. III, 324–25 (HF44-5).

84
*Ibid.*, Vol. II, 67.

85
*Ibid.*, Vol. III, 79 and 324–25 (HF44-5).

86 Hollywood companies: *Ibid.*, Vol. III, 323 (HF53) and 345 (HF65); Hollywood actors Vol. III, 321 (HF42). Intro of Hollywood techniques Vol. III, 317–19 (HF37-39) and Vol IV, 144.

87
*Ibid.*, Vol. III, 341–42 (HF61-62).

88
*Ibid.*, Vol. I, for example, 38, 80, 106.

89
*Ibid.*, 107.

90
*Ibid.*, 109.

91
*Ibid.*, 106. Nichtenhauser does not name the film, and several fly films were produced around this time. The details Nichtenhauser provides suggest it may have been *The Fly Pest.* For more on this and other contemporary fly films, see Bill Marsh , ‘Visual education in the United States and the “Fly Pest” Campaign of 1910’, Historical Journal of Film, Radio and Television, 30 (2010), 21–36 . Gaycken, *op. cit.* (note 68), 143–57. Neither Marsh nor Gaycken refer to Nichtenhauser regarding this film.

92 Nichtenhauser, *History*, *op. cit.* (note 1), Vol. I, Chapter: Early Precursors of Cinematography.

93
*Ibid.*, Vol. IV, 59–94, Suppl. 63 (SF89).

94 Bonah, Cantor, and Laukötter, *op. cit.* (note 5), 21.

95 For his account of his own involvement in the American Film Center, see Nichtenhauser, *History*, *op. cit.* (note 1), Vol. III, 351–56 (HF70-76).

96 Conditions of filmmaking: *Ibid.*, Vol. III, 167–70. Germany: Vol. III, 240. Other themes include pioneers of the use of 16 mm film (Vol. II, 110, 131), its relations to new film technologies such as Kodacolor and Kodachrome (Vol. III, 169; Vol. IV, 96), and problems in the supply of projectors for 16 mm film (Vol. III, 115 and 139).

97
*Ibid.*, Vol. I, 170, Vol. III, 170–71 and 299 (HF19).

98
*Ibid.*, Vol. I, 224. See also Vol. I, 195; Vol. II, 170 Suppl 7; Vol. III, 123 and 167–68; Vol. IV, 185 and esp. 114–24. On the rise of amateurs, see Vol. III, 170–73 and 280. For an exception to his critique of amateur filmmaking: Vol. III, 299–300 (HF19-20).

99
*Ibid.*, Vol. II, 112–13, at 112. See also Vol. I, 141–53; Vol. II, 143; Vol. III, 102–3, 183–85, 206, and 235; Vol. IV, 19.

100
*Ibid.*, Vol. III, 298–99 (HF18-19).

101
*Ibid.*, Vol. III, 259 and 264. See also Vol. III, 317 (HF37).

102
*Ibid.*, Vol. IV, 54.

103
*Ibid.*, Vol. II, 121.

104 See, for example, his discussion of sound in *Ibid.*, Vol. II, 121 (where Nichtenhauser claims the soundtrack should be prepared with the help of film professionals, not agency officials/subject matter specialists); Vol. III, 150.

105
*Ibid.*, Vol. III, 150.

106
*Ibid.*, 160.

107
*Ibid.*, 298–99 (HF18-19).

108
*Ibid.*, Vol. IV, 284.

109
*Ibid.*, 285.

110
*Ibid.*, 286.

111
*Ibid.*, Vol. IV, 298.

112 Robert Stam, *Film Theory: An Introduction* (Oxford: Blackwell, 2000), 27. There is a huge literature on Balázs and his view of film as a new form of language, see, for example, Béla Balázs (and Erica Carter [ed.]), Béla Balázs: Early Film Theory: Visible Man and the Spirit of Film (New York: Berghahn Books, 2010).

113 Nichtenhauser, *History*, *op. cit.* (note 1), Vol. IV, 285–89.

114 Adolf Nichtenhauser, ‘Um die Zukunft der stummen Filmkunst’, *Prager Tagblatt*, Prague (22 August 1929), 5.

115 Adolf Nichtenhauser, ‘Buster Keaton’, *Die Wiener Weltbühne* (12 January1933), 54–55. On how Keaton’s contract with Metro-Goldwyn-Mayer meant that he lost his creative autonomy, see James Curtis , Buster Keaton: A Filmmaker’s Life (New York: Alfred A. Knopf, 2022). On Nichtenhauser’s general concerns about how the introduction of sound technologies might lead to a loss of cinematic expression sound, see: Adolf Nichtenhauser, ‘Um die Zukunft der stummen Filmkunst’, *Prager Tagblatt* (22 August 1929), 5.

116 Adolf Nichtenhauser, ‘Der Kinonarr’, *Die Wiener Weltbühne* (29 December 1932), 440–1.

117 There were other examples of talkies where sound complemented or enhanced the visuals. For example, Nichtenhauser also adored *Trouble in Paradise* (1932 – German-language version *Trubel im Paradies*), a romantic comedy directed by Ernst Lubitsch. Nichtenhauser praised some of the physical acting as great theatrical art. He also lauded the staging and the artistic work that in his view produced the naturalism of the film, the mutedness of language and light, and the precision of the editing. Adolf Nichtenhauser, ‘Trubel im Paradies’, *Die Wiener Weltbühne* (4 May 1933), 562.

118 On the problems of finding good lecturers, see Nichtenhauser, *History*, *op. cit.* (note 1), Vol. II, 58–59.

119 Adolf Nichtenhauser, ‘Planned Health Film Production’ Presented to the ‘Motion Pictures in Health Education’, meeting held at the American Film Center, 11 October 1943, 2, Folder, ‘Nichtenhauser, Adolf (typescripts)’, Nichtenhauser Collection, NLM.

120 Nichtenhauser, *History*, *op. cit.* (note 1), Vol. IV, 119.

121

Hans-Joachim Braun , ‘Hollywood and nothing else? The Americanisation of the German film industry in the Weimar Republic’, in Nick Tiratsoo and Mathias Kipping (Dir.). Americanisation in 20th Century Europe: Business, Culture, Politics, Volume 2 (Lille: Publications de l’Institut de recherches historiques du Septentrion, 2002), 177–191 . Stephen Brockmann , A Critical History of German Film (Rochester: Camden House, 2020).

122 Nichtenhauser, *History*, *op. cit.* (note 1), Vol. II, 114. Also, on the work of Lashley and Watson Vol. II, 82–91. See also, Anja Laukötter , Sex – Richtig! Körperpolitik Und Gefühlserziehung Im Kino Des 20. Jahrhunderts (Göttingen: Wallstein Verlag, 2021).

123 Nichtenhauser, *Ibid.*, Vol. II, 122.

124
*Ibid.*, Vol. I, 105 (films produced for public don’t work in schools); see also Vol. II, 123–24. Paradoxically, Nichtenhauser also quoted work that compared the educational level of the public with that of a grammar school graduate or higher educational level Vol. II, 117–18.

125
*Ibid.*, Vol. II, 117. The text is underlined for emphasis in the original.

126 Nichtenhauser here seems to be building on the ideas of German-speaking reformists of the 1910s and 20s, see Scott Curtis , The Shape of Spectatorship: Art, Science, and Early Cinema in Germany (New York: Columbia University Press, 2015), Chapter 2.

127 Nichtenhauser, ‘Der Aufbau’ and ‘Progress’, *op. cit.* (note 34).

128 Nichtenhauser, ‘Was wird’, *op. cit*. (note 30), at 400.

129

Matthew W. Dunne , A Cold War State of Mind: Brainwashing and Postwar American Society (Amherst: University of Massachusetts Press, 2013). Daniel Pick , Brainwashed: A New History of Thought Control (London: Wellcome Collection, 2023).

130 Nichtenhauser, *History*, *op. cit.* (note 1), Vol. III, 9. (Quoting Curt Thomalla regarding *The Curse of Heredity* [1927])

131
*Ibid.*, 46. (Quoting André Cavaillon)

132 Nichtenhauser, *op. cit.* (note 23).

133 David S. Ruhe to Adolf Nichtenhauser 13 December 1946, Folder, ‘Navy Med. Film Project, 1945-1947’, Nichtenhauser Collection, NLM.

134
*Ibid.*

135
*Ibid.*

136 In the case of his tendency to hide the intellectual Viennese roots of his interest in film, and visual education and language, he likely also wanted to ensure that the reader was not bogged down with detail. His story of how he became interested in film was among documents he used to apply for jobs, so likely not a place for his intellectual roots, given the leftish politics of some of the circles he moved in in Vienna, and the need to avoid losing non-specialists in intellectual detail.

137 Such concerns are likely behind Owsei Temkin’s comments about the decline in classical learning and the inability of persons to enter into worlds quite different from that of their own. See his ‘The Study of the History of Medicine,’ in Owsei Temkin, *On Second Thought And Other Essays in the History of Medicine and Science* (Baltimore and London: Johns Hopkins University Press, 2002), 231–40, esp. 231. Essay first published in 1959. See David Cantor, Essay review of Owsei Temkin, ‘*“On Second Thought” And Other Essays in the History of Medicine and Science*, Baltimore: Johns Hopkins University Press, 2002’, *Theoretical Medicine and Bioethics*, 25 (2004), 157–164.

138 Nichtenhauser, *History*, *op. cit.* (note 1), Vol. I, p.XII.

139 MOMA offered his research papers, files, and correspondence to the National Library of Medicine in 1954. Sometime after 1979 MOMA transferred Nichtenhauser’s typescript history and accompanying photographs and negatives to the Naval Health Sciences Education and Training Command. The typescript was given to NLM in 1981, while the photographs and a photostat of the typescript were transferred to the historian of medicine Robert J.T. Joy, M.D., of the Uniformed Services University of the Health Sciences. In 1991, Joy gave the photographs and negatives to the NLM. Provenance: Gift, Museum of Modern Art, 1954. Gift, Malcolm S. Ferguson (Naval Health Sciences Education and Training Command), 12/8/1981, Accession #328. Gift, Robert T. Joy (USUHS), 3/8/1991, Accession #665.

140 For a surprising example of a recent book that does not cite Nichtenhauser given his interest in central European film, see Victoria Shmidt and Karl Kaser , A Critical History of Health Films in Central and Eastern Europe and Beyond (London and New York: Routledge, 2024).

141 Thus, for example, he has been cited for his insights into the early history of scientific film, the origins of the public health education film in the 1910s, the early history of audience research, and even his interest in visual language of medicine, along with his published works, especially on mental health film.

142 An exception is Michael Sappol, ‘The Public Health Film Goes to War’, 27 March 2023 (originally published, 2011), https://medicineonscreen.nlm.nih.gov/2023/03/27/the-public-health-film-goes-to-war/ (accessed 23 March 2025).

143 For an exception, see Christian Bonah and Anja Laukötter , ‘Introduction: Screening Diseases. Films on Sex Hygiene in Germany and France in the First Half of the 20th Century’, Gesnerus, 72 (2015), 5–14 26403052 .

